# Association between lithium use and the incidence of dementia and its subtypes: A retrospective cohort study

**DOI:** 10.1371/journal.pmed.1003941

**Published:** 2022-03-17

**Authors:** Shanquan Chen, Benjamin R. Underwood, Peter B. Jones, Jonathan R. Lewis, Rudolf N. Cardinal

**Affiliations:** 1 Department of Psychiatry, University of Cambridge, Cambridge, United Kingdom; 2 Cambridgeshire and Peterborough NHS Foundation Trust, Cambridge, United Kingdom; 3 NIHR Applied Research Collaboration, East of England, Cambridge, United Kingdom; Harvard Medical School, UNITED STATES

## Abstract

**Background:**

Dementia is the leading cause of death in elderly Western populations. Preventative interventions that could delay dementia onset even modestly would provide a major public health impact. There are no disease-modifying treatments currently available. Lithium has been proposed as a potential treatment. We assessed the association between lithium use and the incidence of dementia and its subtypes.

**Methods and findings:**

We conducted a retrospective cohort study comparing patients treated between January 1, 2005 and December 31, 2019, using data from electronic clinical records of secondary care mental health (MH) services in Cambridgeshire and Peterborough NHS Foundation Trust (CPFT), United Kingdom (catchment area population approximately 0.86 million). Eligible patients were those aged 50 years or over at baseline and who had at least 1 year follow-up, excluding patients with a diagnosis of mild cognitive impairment (MCI) or dementia before, or less than 1 year after, their start date. The intervention was the use of lithium. The main outcomes were dementia and its subtypes, diagnosed and classified according to the International Classification of Diseases-10th Revision (ICD-10).

In this cohort, 29,618 patients (of whom 548 were exposed to lithium) were included. Their mean age was 73.9 years. A total of 40.2% were male, 33.3% were married or in a civil partnership, and 71.0% were of white ethnicity. Lithium-exposed patients were more likely to be married, cohabiting or in a civil partnership, to be a current/former smoker, to have used antipsychotics, and to have comorbid depression, mania/bipolar affective disorder (BPAD), hypertension, central vascular disease, diabetes mellitus, or hyperlipidemias. No significant difference between the 2 groups was observed for other characteristics, including age, sex, and alcohol-related disorders. In the exposed cohort, 53 (9.7%) patients were diagnosed with dementia, including 36 (6.8%) with Alzheimer disease (AD) and 13 (2.6%) with vascular dementia (VD). In the unexposed cohort, corresponding numbers were the following: dementia 3,244 (11.2%), AD 2,276 (8.1%), and VD 698 (2.6%). After controlling for sociodemographic factors, smoking status, other medications, other mental comorbidities, and physical comorbidities, lithium use was associated with a lower risk of dementia (hazard ratio [HR] 0.56, 95% confidence interval [CI] 0.40 to 0.78), including AD (HR 0.55, 95% CI 0.37 to 0.82) and VD (HR 0.36, 95% CI 0.19 to 0.69). Lithium appeared protective in short-term (≤1-year exposure) and long-term lithium users (>5-year exposure); a lack of difference for intermediate durations was likely due to lack of power, but there was some evidence for additional benefit with longer exposure durations. The main limitation was the handling of BPAD, the most common reason for lithium prescription but also a risk factor for dementia. This potential confounder would most likely cause an increase in dementia in the exposed group, whereas we found the opposite, and the sensitivity analysis confirmed the primary results. However, the specific nature of the group of patients exposed to lithium means that caution is needed in extending these findings to the general population. Another limitation is that our sample size of patients using lithium was small, reflected in the wide CIs for results relating to some durations of lithium exposure, although again sensitivity analyses remained consistent with our primary findings.

**Conclusions:**

We observed an association between lithium use and a decreased risk of developing dementia. This lends further support to the idea that lithium may be a disease-modifying treatment for dementia and that this is a promising treatment to take forwards to larger randomised controlled trials (RCTs) for this indication.

## Abstract 2

Shanquan Chen and colleagues investigate the association between lithium use and the incidence of dementia and its subtypes.

## Author summary

**Why was this study done?:**

- Dementia is the leading cause of death and disability in elderly Western populations.
- Preventative interventions that could delay dementia onset even modestly would provide a major public health impact.
- Lithium has been proposed as a potential treatment, but limited population-level research has been conducted to support the idea that lithium might delay the onset of dementia.

**What did the researchers do and find?:**

- We identified 548 patients who were exposed to lithium and 29,070 patients who were not exposed to lithium between January 1, 2005 and December 31, 2019, using data from electronic clinical records of secondary care mental health (MH) services in Cambridgeshire and Peterborough NHS Foundation Trust (CPFT), UK.
- We assessed the association between lithium use and the incidence of dementia and its subtypes, including Alzheimer disease (AD) and vascular dementia (VD).
- We found that lithium was associated with a lower risk of receiving a diagnosis of dementia, including a lower risk of being diagnosed with either AD or VD.
- The extended analysis by the duration of lithium treatment indicated that both short- (≤1-year exposure) and long-term (>5-year exposure) lithium exposure (but not medium term, 1- to 5-year lithium exposure) was associated with decreased incidence of dementia and its subtypes. Power was less for the intermediate durations. There was some evidence for additional benefit with longer exposure durations.

**What do these findings mean?:**

- Our findings support the possibility that lithium treatment could decrease the risk of developing dementia and supports the need for further randomised controlled trials (RCTs) to test the efficacy of lithium as a disease-modifying drug in dementia.
- The main limitation is that 73% of the patients in lithium-exposed group had mania/bipolar affective disorder (BPAD), which is a significant risk factor for dementia. Our results were in the opposite direction, however, and were supported by sensitivity analyses.

## Introduction

Dementia is the leading cause of death and disability in elderly Western populations: Approximately 47 million people had dementia worldwide in 2015, and this number is projected to triple by 2050 [1]. Preventative interventions that could delay dementia onset even modestly would provide a major public health impact. It has been estimated that delaying onset by 5 years would reduce the prevalence and economic impact of dementia by 40% [2]. Lithium has been proposed as a potential therapy; it has positive effects in cell and animal models of dementia [3–5], and there is evidence for its neuroprotective effects from experimental research and clinical studies using brain imaging [6–8]. The putative neuroprotective mechanism of action of lithium is not known, but may include tau phosphorylation, up-regulation of autophagy, antiapoptotic effects, and triggering of neurotrophic cascades [9]. Two meta-analyses [10,11] and a subsequent randomised controlled trial (RCT) [12] have suggested that lithium has beneficial effects on cognitive performance in mild cognitive impairment (MCI) and Alzheimer disease (AD). However, it is unclear whether lithium can delay the onset of overt dementia, and lithium has been reported as a risk factor [13,14] as well as a protective factor [15–19] for developing dementia.

One major consideration in observational studies is the need to account for the influence of mania/bipolar affective disorder (BPAD), or unipolar depression, the 2 most common indications for lithium. BPAD and depression may both themselves increase the risk of dementia [3,11,20]. Some previous studies have been large [13,14,16–19], but did not control for concomitant other medications (except for psychotropics [16,18,19]) and morbidities [13,17–19], did not take into consideration the cumulative risk of time [3,17], or their follow-up duration was short compared with the time course of the onset of dementia [14,16]. A 2019 review [3] emphasised that the relationship between lithium use and the incidence of subtypes of dementia (as opposed to “dementia” as a group of disorders) has not been thoroughly investigated. Three studies from Denmark [18,19] and the United States of America [16] implied that there may be a cumulative effect for lithium over time in terms of modifying the risk of developing dementia. The available evidence for this possible cumulative effect is based on lithium prescriptions or short duration of lithium exposure of no more than 1 year. Evidence in relation to longer-term lithium exposure is needed.

In this study, our primary aim was to assess the association of lithium use with the incidence of dementia and its subtypes via a retrospective cohort study, with analysis of potential confounding factors, in routinely collected clinical data covering a 15-year period. Our second aim was to examine the degree of association by the duration of lithium treatment. We hypothesised that lithium would be associated with a reduced risk of dementia and its subtypes, and this association would be seen in both short- and long-term lithium exposure.

## Methods

### Study design and participants

We performed a retrospective cohort study using data from the electronic clinical records of Cambridgeshire and Peterborough NHS Foundation Trust (CPFT), UK, deidentified to create the CPFT Research Database [21]. CPFT provides community and mental health (MH) services to a population of approximately 0.86 million people (37.3% of whom are aged 50 years or over, compared with the national figure of 37.9% for England), covering both urban (the primary composition) and rural areas. The present study examined those referred at some point to secondary care MH services. The MH electronic record contains patient information recorded by clinicians during routine treatment, including structured data (e.g., age, sex, marital status, ethnicity, and coded diagnoses) and unstructured data (free-text notes, semistructured textual forms, and text from binary documents) [22]. Routine blood tests for lithium, where recorded in the clinical notes, provide a measure of concordance.

We examined data between January 1, 2005 and December 31, 2019. For the cohort of patients exposed to lithium (group Li+), each patient’s origin time (start date) was defined as their earliest recorded exposure to lithium (defined below). For the unexposed cohort (group Li−), the origin time was the latest of their CPFT registration date or January 1, 2005. Follow-up was until the patients’ final record, death, or the first record of dementia, whichever occurred first. Eligible patients were those aged ≥50 years at baseline and had at least 1 year of follow-up. We excluded patients with a preexisting diagnosis of MCI or dementia or those diagnosed <1 year after their origin time.

### Data collection

The CPFT Research Database operates on an opt-out basis and contains data from most CPFT patients (at present totalling approximately 270,000 across all ages). Natural language processing (NLP) tools were used to extract smoking status, medication [23], and lithium blood test information [21] from unstructured text.

#### Outcomes and measures

Dementia was diagnosed and classified according to the World Health Organization’s (WHO) International Classification of Diseases-10th Revision (ICD-10), using codes F00 (AD), F01 (vascular dementia, VD), F02 (dementia in other diseases), F03 (unspecified dementia), and G30 (AD). We also subdivided dementia into AD (F00, G30) and VD (F01) as independent outcomes.

#### Exposure

Exposure to lithium was judged if patients were prescribed lithium (via NLP tools detecting medication mentions) or had a serum lithium level of ≥0.2 mmol/L recorded (via NLP tools detecting lithium test results). The duration of lithium exposure was calculated as the range between the earliest and latest date of lithium events (defined as above) and was treated as a categorical variable. There is no uniform standard to categorise lithium exposure duration; we chose 1 year as the minimum duration of treatment likely to have a function on dementia outcomes [3,12]. We further divided exposure into categories: no exposure, <1 year, 1 to 2 years, 2 to 3 years, 3 to 4 years, 4 to 5 years, and >5 years (throughout, the lower limit of each range is exclusive, and the upper limit is inclusive).

#### Covariates

We investigated several sociodemographic variables: age at baseline (years), sex (male versus female), marital status (married, cohabiting, or civil partnership versus not), and ethnicity (white versus others/unknown). We investigated smoking status (current or past smoker versus not). We also investigated coprescription and comorbidity as potential confounders and treated them as binary time-constant variables (i.e., whether the medications had ever been prescribed to the patient or whether they had ever had the condition). Medication/comorbidity variables were the following: taking **antipsychotics** (yes or no) (see Table A in S1 Appendix); **depression** (defined as the presence of ICD-10 codes F32 [depressive episode] or F33 [recurrent depressive disorder] or taking antidepressant medications); **alcohol-related disorders** (ICD-10 codes starting F10); **mania or BPAD** (ICD-10 codes F30 [mania] or F31 [BPAD] or taking medications carbamazepine, lamotrigine, and valproate [including semisodium valproate]); **diabetes mellitus** (ICD-10 codes E10 to E14 or taking hypoglycemic agents); **hypertension** (ICD-10 codes I10-I13 or I15 or taking antihypertensive medications); **central (coronary or cerebral) vascular disease** (ICD-10 codes I21 to I25 and I60 to I69 or taking ACE inhibitors, angiotensin II receptor antagonists, beta-blockers, calcium channel antagonists, or diuretics); and **hyperlipidemias** (ICD-10 codes E78 or taking lipid-lowering medications). Identification of these concomitant medications or morbidities was based on CPFT records up to 1 year before the end of follow-up. The medicines referred to were selected according to UK National Institute for Health and Care Excellence (NICE) guidelines (Table A in S1 Appendix).

#### Other variables

MCI was identified with ICD-10 codes starting F06.7. Death was ascertained by weekly linkage to national NHS Spine mortality data for all patients known to CPFT MH services.

### Statistical analysis

We report categorical variables as number (percentage) and continuous variables as mean (standard deviation). Differences between exposure groups were assessed via 2-tailed *t* tests (for continuous variables) and chi-squared tests (for categorical variables).

Cox proportional hazard models were used to examine the corrected association between lithium exposure and risk of incident dementia, covarying for sociodemographic variables, smoking status, coprescription, and comorbidities. Unlike RCTs, clinical decisions to prescribe lithium are often based on prognostic factors. Therefore, the association estimate for lithium might be confounded by treatment selection. To further control for possible selection bias between lithium exposure and nonexposure, the Cox models were adjusted by inverse probability weights (IPWs). IPW (or inverse probability of treatment weighting) is an extension of the propensity score method used to summarise the conditional probability of assignment for a treatment [24,25]. We derived IPWs from propensity scores generated by a binary logistic regression model of lithium exposure, with the same covariates included as in the Cox models. We report group comparisons prior to IPW and standardised mean differences (SMDs) between groups (SMD = mean difference divided by the standard deviation of the variable) before and after IPW, including as a measure of balance before/after weighting.

We repeated the Cox regression to analyse associations by duration of lithium exposure.

Data were complete for outcomes and predictors except for ethnicity and any unmeasured diagnostic undercoding in clinical practice. We used multiple imputation with chained equations [26] and generated 5 imputed data sets to reduce bias and maintain power.

We compared models using partial likelihood ratio tests (as models were not nested) and present also Akaike information criterion (AIC) values.

We performed sensitivity analyses as follows. (1) We used a longer criterion (up to 2 years before the end of follow-up) to identify concomitant medications or morbidities. Since the follow-up duration was long and medications/morbidities were treated as constant (time-invariant) variables, then (for example) if hypertension develops only in the last year of follow-up, it would be treated as present throughout for that patient. This sensitivity analysis tests the potential bias of including late-onset medication/conditions or not. (2) We required a longer follow-up (at least 2 years), in order to exclude patients seen only transiently (e.g., registered in CPFT temporarily, such as visitors, or people discharged after a single assessment). (3) We added the assumption that all lithium users had BPAD, to account for diagnostic undercoding of BPAD. (The primary UK indications for lithium are mania/BPAD and recurrent depression. BPAD is a more common indication; note also that the use for unipolar depression is off-licence e.g., in the USA [27]). (4) We excluded everyone diagnosed with recurrent depression (the other main indication for lithium). (5) We excluded ethnicity as a predictor. (6) To consider the possible competing effects between death and dementia, instead of Cox proportional hazard models, we adopted Fine and Gray’s [28] competing risks regression models, incorporating death as a competing event for incident dementia/subtypes. (7) We repeated our analysis controlling only for confounders known at baseline. (8) We repeated our analyses treating exposure to lithium and the duration of exposure to lithium as time-varying variables, respectively. (9) We pooled intermediate exposure durations, creating a single intermediate (1 to 5 years) group. (10) We added the interaction term lithium exposure × duration of exposure, to measure the effects of exposure duration over and above the fact of exposure.

Analyses were performed using R (v3.5.0), including the packages survival (v3.1.7), ipw (v1.0.11), timereg (v1.9.4), dplyr (v0.8.0.1), mice (v3.11.0), weights (v1.0.4), forestplot (v1.9), and nonnestcox (v0.0.0.9). Statistical significance was defined as *p* < 0.05; all tests were 2-tailed. Adjusted survival curves (resembling Kaplan–Meier plots but adjusted for IPW) were generated using the RISCA (v0.9) package.

### Planning of analyses

The analysis plan was made prior to the start of all analyses and agreed upon among coauthors. No data-driven changes to the analysis plan were made. Four additional sensitivity analyses were included in response to peer review, including controlling only for confounders known at baseline, treating the duration of exposure to lithium as a time-varying variable, pooling intermediate exposure durations, and adding the lithium exposure × exposure duration interaction.

This study is reported following the Strengthening the Reporting of Observational Studies in Epidemiology (STROBE) guideline (S1 Checklist) [29].

### Ethics declarations

#### Ethics approval and consent to participate

Deidentified data were extracted from the electronic clinical records of Cambridgeshire and Peterborough NHS Foundation Trust (CPFT) under NHS Research Ethics approvals (references 12/EE/0407 and 17/EE/0442). No patients were involved in the development of the research question or the outcome measures or in developing plans for the design and analysis of the study.

## Results

**Fig 1:**
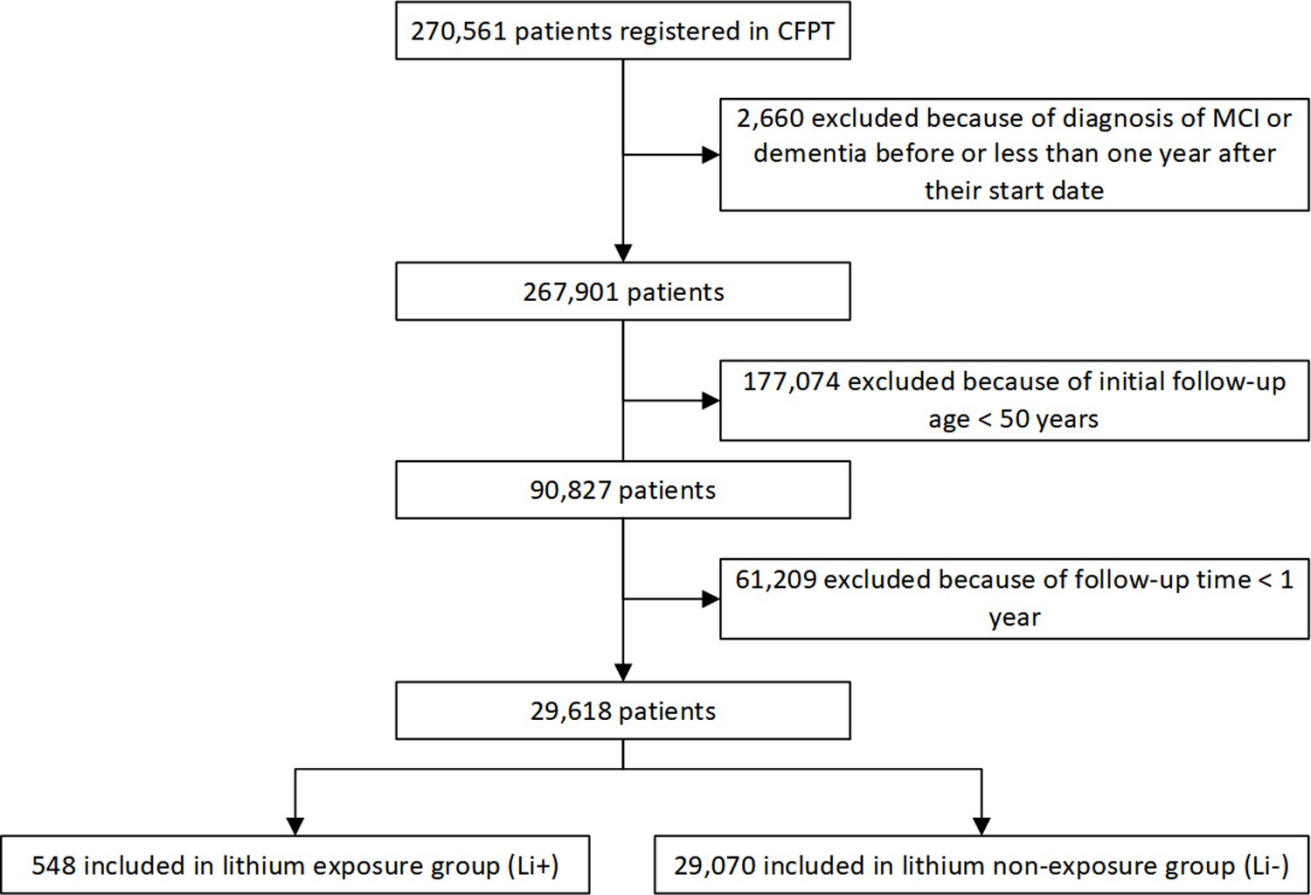
STROBE diagram showing construction of the cohorts. CPFT, Cambridgeshire and Peterborough NHS Foundation Trust; MCI, mild cognitive impairment; STROBE, Strengthening the Reporting of Observational Studies in Epidemiology.

We followed 548 patients exposed to lithium (Li+) and 29,070 patients unexposed to lithium (Li−) (Fig 1). The cohorts were followed up for a mean of 4.8 years (ranging from 1 to 14.9 years) and 4.3 years (ranging from 1 to 14.6 years), respectively (Table 1). In more detail, those exposed to lithium (Li+) for <1 year were followed up for a mean of 4.2 years, those exposed for 1 to 2 years for 2.68 years, those exposed 2 to 3 years for 2.75 years, those exposed 3 to 4 years for 2.51 years, those exposed 4 to 5 years for 3.47 years, and those exposed for >5 years for 6.54 years. There were some differences in the baseline characteristics of patients between the 2 cohorts (Table 1). Patients in the Li+ cohort were more likely to be married, cohabiting, or in a civil partnership (*p* < 0.001), to be a current/former smoker (*p* < 0.0001), to have used antipsychotics (*p* < 0.0001), and to have comorbid depression (*p* < 0.0001), BPAD (*p* < 0.0001), hypertension (*p* < 0.0001), central vascular disease (*p* < 0.0001), diabetes mellitus (*p* < 0.0001), and hyperlipidemias (*p* < 0.0001). With the exception of marriage, all these factors predict an increased, not decreased, risk of dementia. No significant difference between the 2 groups was observed for other characteristics, including age (*p* = 0.0857), sex (*p* = 0.760), and alcohol-related disorders (*p* = 0.8666). After IPW adjustment, the baseline characteristics between cohorts were well balanced, except for having comorbid BPAD (SMD = 0.4208). Across all NLP-derived records of lithium plasma levels, the mean lithium level was 0.61 mM, and the interquartile range was 0.49 to 0.80 mM.

**Table 1:** Patient characteristics.

	Exposed group (Li+)	Unexposed group (Li−)	*p*-Value	SMD before IPW	SMD after IPW
	(*n* = 548)	(*n* = 29,070)			
**Age (years) at entry**	73.05 (11.82)	73.93 (12.72)	0.0857	0.0004	0.0003
**Sex (male)**	224 (40.9)	11,668 (40.1)	0.7601	0.0150	0.0021
**Marital status**					
Married/civil partnership	231 (42.2)	9,634 (33.1)	<0.0001	0.4210	0.0860
Cohabiting	2 (0.4)	52 (0.2)			
Single	69 (12.6)	1,881 (6.5)			
Divorced/separated	48 (8.8)	3,140 (10.8)			
Widowed	62 (11.3)	5,791 (19.2)			
Unknown	136 (24.8)	8,576 (29.5)			
**Ethnicity**					
White	409 (74.6)	20,616 (70.9)	0.0005	0.1670	0.0930
Asian	12 (2.2)	233 (0.8)			
Black	3 (0.5)	87 (0.3)			
Other	57 (10.4)	3,790 (13)			
Not known	67 (12.2)	4,344 (14.9)			
**Smoker (current or former)**	34 (6.2)	699 (2.4)	<0.0001	0.1880	0.0762
**Alcohol-related disorder (yes)**	6 (1.1)	271 (0.9)	0.8666	0.0160	0.0330
**Antipsychotic use (yes)**	230 (42)	1,998 (6.9)	<0.0001	0.8950	0.0371
**Depression (yes)**	355 (64.8)	6,090 (20.9)	<0.0001	0.9010	0.0830
**Mania or BPAD (yes)**	399 (72.8)	971 (3.3)	<0.0001	0.9880	0.4208
**Hypertension (yes)**	154 (28.1)	3,915 (13.5)	<0.0001	0.3670	0.0523
**Central vascular disease (yes)**	236 (43.1)	5,681 (19.5)	<0.0001	0.5240	0.0813
**Diabetes mellitus (yes)**	50 (9.1)	987 (3.4)	<0.0001	0.2380	0.0402
**Hyperlipidemias (yes)**	140 (25.5)	3,082 (10.6)	<0.0001	0.3960	0.0021
**Duration of lithium exposure**					
No exposure		29,070 (100)			
>0, < = 1 years	323 (58.9)				
>1, < = 2 years	47 (8.6)				
>2, < = 3 years	32 (5.8)				
>3, < = 4 years	22 (4)				
>4, < = 5 years	23 (4.2)				
>5 years	101 (18.4)				
**Number who developed dementia during follow-up**					
Dementia (yes)	53 (9.7)	3,244 (11.2)	0.2776	0.0490	0.1760
AD (yes)	36 (6.8)	2,276 (8.1)	0.2613	0.0500	0.1330
VD (yes)	13 (2.6)	698 (2.6)	0.8612	0.0050	0.2370
**Time to event (years)**	4.77 (3.32)	4.31 (2.89)	<0.0001	0.0008	0.0114

Data are shown as mean (SD) for age and time to event or number (percentage) for others. *p*-Values reflect unweighted group comparison; for age and time to event, they were obtained by *t* test and for others via Pearson chi-squared test.

AD, Alzheimer disease; BPAD, bipolar affective disorder/mania; IPW, inverse probability weighting; SMD, standardised mean difference between groups; VD, vascular dementia.

**Fig 2:**
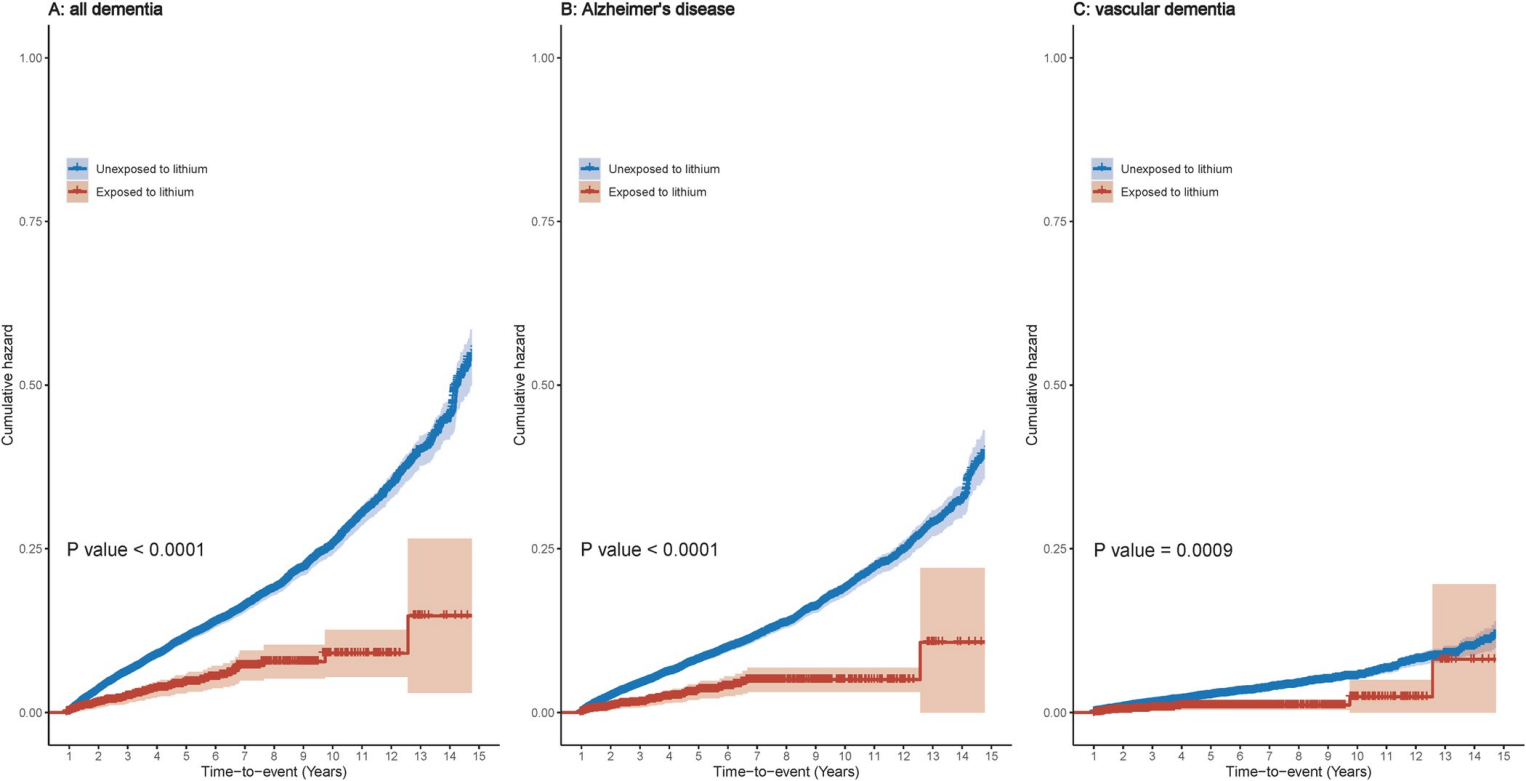
Cumulative hazard of dementia and its subtypes in lithium users versus nonusers. *p*-Values are calculated from the log-rank test after IPW. IPW, inverse probability weights/weighting.

In the exposed cohort, there were 53 (9.7%) patients diagnosed with dementia, including 36 (6.8%) with AD and 13 (2.6%) with VD. The cumulative hazard of dementia and its subtypes in lithium users versus nonusers is shown in Fig 2: Lithium was associated with a significantly lower risk of dementia and its subtypes (*p*-values < = 0.0009).

**Fig 3:**
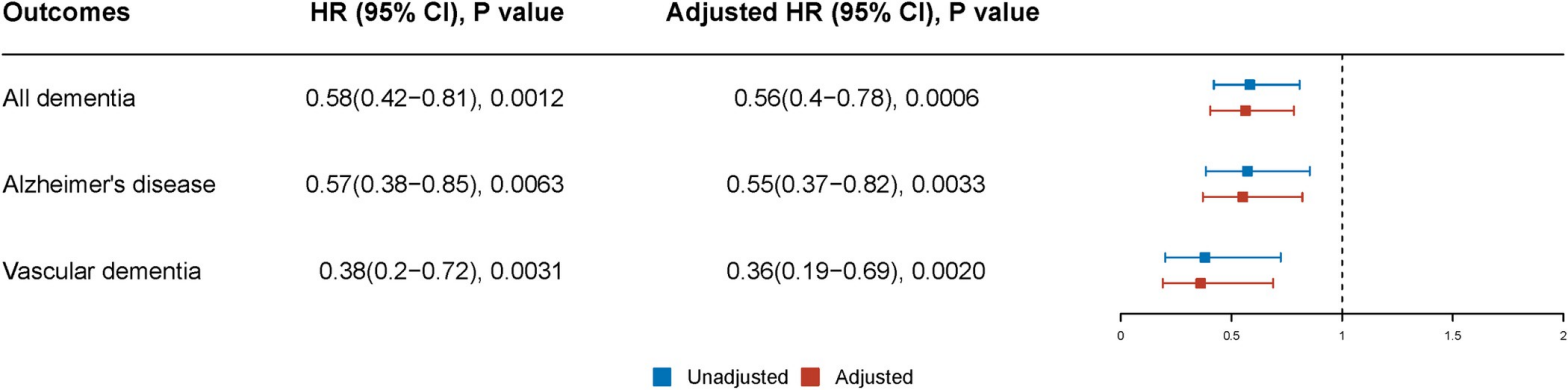
HRs, 95% CIs, and *p*-values for the association of lithium with the development of dementia (and its subtypes) by Cox proportional hazards models. HRs, 95% CIs, and *p*-values were extracted from inverse probability weighted Cox regression. Adjusted HRs were adjusted for age, sex, marital status, ethnicity, smoking status, alcohol disorders, antipsychotic use, depression, mania or BPAD, hypertension, central vascular disease, diabetes mellitus, and hyperlipidemias. The AIC was 14,275 for the model fitted for all dementias, 13,240 for the model fitted for AD, and 13,220 for the model fitted for VD. AD, Alzheimer disease; AIC, Akaike information criterion; BPAD, bipolar affective disorder/mania; CI, confidence interval; HR, hazard ratio; VD, vascular dementia.

**Fig 4:**
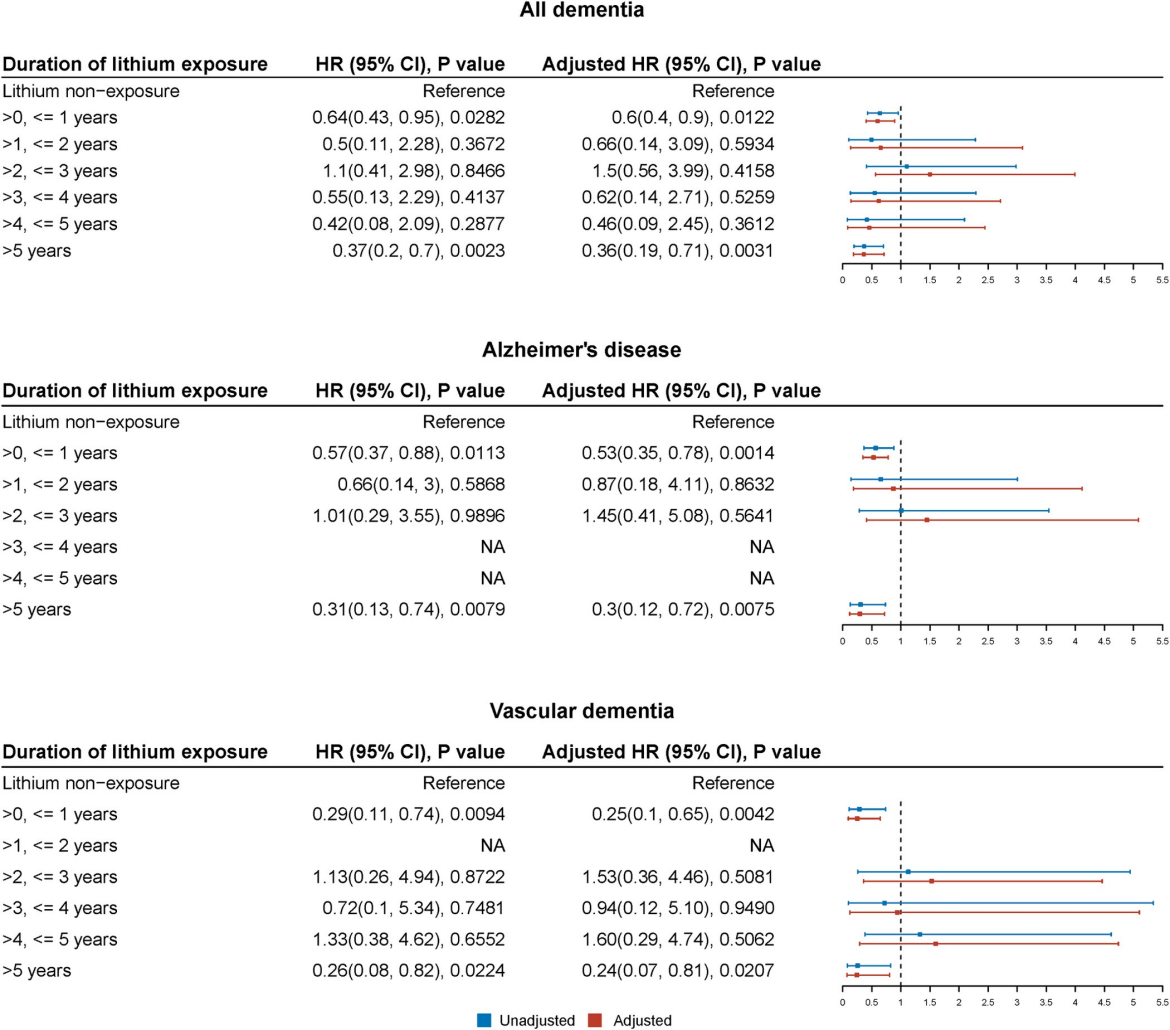
HRs, 95% CIs, and *p*-values for the association of duration of lithium exposure with the development of dementia (and its subtypes) by Cox proportional hazards models. HRs, 95% CIs, and *p*-values were extracted from inverse probability weighted Cox regression. Adjusted HRs were adjusted for age, sex, marital status, ethnicity, smoking status, alcohol disorders, antipsychotic use, depression, mania or BPAD, hypertension, central vascular disease, diabetes mellitus, and hyperlipidemias. NA indicates no result (no corresponding cases). The AIC was 14,125 for the model fitted for all dementias, 13,064 for the model fitted for AD, and 13,119 for the model fitted for VD. See Fig Q in S1 Appendix for an equivalent analysis using pooled intermediate durations and Fig R in S1 Appendix for the effects of lithium exposure duration as a continuous variable. AD, Alzheimer disease; AIC, Akaike information criterion; BPAD, bipolar affective disorder/mania; CI, confidence interval; HR, hazard ratio; VD, vascular dementia.

After controlling for confounding by sociodemographic factors (age, sex, marital status, and ethnicity), smoking status, other medications, other mental comorbidities, and physical comorbidities, compared with patients who never took lithium, exposure to lithium was associated with a lower risk of dementia (adjusted hazard ratio [HR] 0.56, 95% confidence interval [CI] 0.40 to 0.78; *p* = 0.0006) and its subtypes, including AD (adjusted HR 0.55, 95% CI 0.37 to 0.82; *p* = 0.0033) and VD (adjusted HR 0.36, 95% CI 0.19 to 0.69; *p* = 0.002) (Fig 3). Analyses by duration of lithium exposure (Fig 4) suggested that lithium was associated with a reduction in dementia (collectively, or AD, or VD) at short-term (≤1 year) exposure and long-term (>5 years) exposure. Effects at intermediate durations were not significant; however, contributory numbers were smaller for intermediate durations (Table 1) (or even absent in some cases for subtypes of dementia) and CIs were wide. The models stratified by exposure duration (Fig 4) were a slightly better fit than the basic, unstratified models (Fig 3) (*p* < 0.01).

Sensitivity analyses using a longer medication/comorbidity discovery criteria (Figs A and B in S1 Appendix), a longer follow-up (Figs C and D in S1 Appendix), treating all lithium users as having BPAD (Figs E and F in S1 Appendix), excluding those with recurrent depression (Figs G and H in S1 Appendix), excluding ethnicity as a predictor (Figs I and J in S1 Appendix), considering competing effects from death (Figs K and L in S1 Appendix), only controlling for confounders known at baseline (Figs M and N in S1 Appendix), treating exposure to lithium (yes or no) and the duration of exposure to lithium as time-varying variables (Figs O and P in S1 Appendix), or pooling intermediate exposure durations (Fig Q in S1 Appendix) all confirmed our primary results that lithium was associated with a lower risk of dementia and its subtypes (AD and VD). As before, this association was seen for short-term exposure (≤1 year) and long-term exposure (>5 years), but was not significant for medium-term exposure durations (again, likely related to lower power for intermediate durations).

Analysis of the effects of exposure duration, over and above the fact of exposure, suggested a significant decrease in risk associated with additional exposure to lithium, for all dementias and AD (Fig Q in S1 Appendix), although this duration-related effect was not significant for VD, for which power was lowest (Table 1).

## Discussion

Using a retrospective cohort study based on a large and comprehensive clinical record database, and after controlling for sociodemographic factors (age, sex, marital status, and ethnicity), smoking status, comedications, co-mental morbidities, and co-physical morbidities, we found that lithium was associated with a lower risk of dementia and its subtypes (AD and VD). Analysis by the duration of lithium treatment indicated that both short (≤1-year exposure) and long-term (>5-year exposure) lithium exposure were associated with a decreased incidence of dementia; effects at intermediate durations were not significant, likely due to lesser power (discussed further below), but there was evidence that the risk of dementia reduced further with longer durations of lithium exposure.

Our findings supported the hypothesis that lithium is associated with a reduced risk of dementia and its subtypes. These associations are consistent with a hypothesised protective effect of lithium on dementia from cellular and rodent models [3–5] and generally consistent with studies of clinical data from Brazil [15], the USA [16], and Denmark [17–19], but less consistent with another UK study [13] and one study conducted in Taiwan [14]. Differences in sample selection, study design, and covariates may account for this inconsistency [3]. For instance, despite the large sample size in the study conducted in Taiwan [3], it did not take into consideration the cumulative risk of time, and the primary analyses did not take into account confounding by indication for lithium (i.e., prescribing for bipolar disorder or unipolar depression, both being associated with an increased risk of dementia). We examined patients known to secondary care MH services for a prolonged period (1 to 14.9 years) and adjusted for potential confounding sociodemographic factors plus psychiatric and major physical comorbidities. We reduced possible bias by adjusting our statistical model (via IPW) to control for other potential baseline differences between lithium-exposed and nonexposed groups and used sensitivity analyses to ensure consistency of results. RCTs are the best way to test the effect of lithium on the incidence of dementia, but RCTs in this area are hampered by late and variable age of onset of dementia (with a long prodrome), uncertain risk for each individual, the requirement for long follow-up, and significant financial costs of these studies [30,31].

To the best of our knowledge, our study is the first to examine the association between lithium and VD in people prescribed lithium. Two studies from Denmark [18,19] have commented on VD, but did not separate VD from other subtypes of dementia except AD. In our study, we found that lithium treatment was associated with a reduced risk of receiving a diagnosis of VD, which suggests any protective effect may be general rather than disease specific. Future work is needed to evaluate the association between lithium and other dementia subtypes, such as Lewy body dementia and Parkinson disease dementia. One previous case–control study [17] reported that high lithium exposure in drinking water was associated with a lower incidence of VD than low exposure, although with nonlinear associations and higher rates at intermediate exposure. Although lithium levels were not consistently available in our study, they lay generally within the established therapeutic range (plasma levels of 0.4 to 1.0 mM), much higher than lithium levels in drinking water (2 to 27 μg/L = 0.29 to 3.86 μM) [17]. Our results extend existing data and suggest that exposure to lithium at standard clinical dosages is associated with a reduced incidence of VD.

While the potential for lithium to slow progression in AD has been studied experimentally [3], to the best of our knowledge, the association of therapeutic lithium with a reduction in VD is novel. However, in animal models, lithium is known to be protective against vascular injury [32] and to decrease atherosclerotic plaque formation [33] and plaque macrophage load [34]; thus, a protective effect for VD is biologically plausible. Likewise, a reduction in VD (as for AD) was seen in relation to high levels of lithium levels in drinking water in Denmark [17]. Of course, the diagnoses recorded in the present data study were clinical and not postmortem diagnoses. It is possible that those diagnosed with AD and/or VD in fact had the other condition or mixed dementia; however, the strength of the association seen for both disorders was not suggestive of a strongly differential association.

We have further expanded on the existing literature by examining the influence of the duration of lithium treatment, as previously suggested [16,35,36], and found that both short-term (<1 year) and long-term (>5 years) exposure to lithium was associated with a decreased risk of dementia. This short-term association is to some extent consistent with another 4-year retrospective cohort study based on 41,931 patients with BPAD [16], which concluded that patients exposed to lithium for 301 to 365 days (but not shorter periods) had a lower risk of dementia than nonexposed patients, as well as with another 2 RCTs [36,37], showing that lithium improved global cognitive performance in patients with MCI or early AD after 10 weeks or 12 months follow-up. The possible long-term protective function of lithium in our study is also consistent with a retrospective study of 4,856 patients with manic/mixed affective episodes or BPAD [19], in which patients prescribed lithium at least 20 times had a lower risk of dementia than those prescribed lithium once.

In our study, medium-term treatment with lithium (1 to 5 years) was not associated with a significantly decreased incidence of dementia. A true U-shaped or bimodal association of duration would be hard to explain if the function of lithium on cognitive function is cumulative and chronic [12,36,37]. Indeed, our data do not provide evidence for a U-shaped effect (e.g., Figs P-R in S1 Appendix). Instead, the power to detect associations at these medium exposure times was limited in comparison to short- and long-term use, largely due to low patient numbers (Table 1, Fig 3). This was likely because lithium is used for prolonged periods when it is tolerated and effective or abandoned early if it is not. In addition, the relatively short follow-up for patients exposed to lithium for 1 to 5 years could have contributed to lower statistical power. Our results do not exclude the possibility of undetected associations for medium-term use. A previous study has also raised this possibility, finding that patients prescribed lithium 5 to 19 times had a lower risk of dementia than those prescribed lithium once [19]. Additionally, exposure-stratified models were a better fit than unstratified models, suggesting changes by exposure duration, and a direct examination of this effect suggested a linear, duration-dependent reduction in the risk of all-cause dementia or AD with longer lithium exposure (Fig R in S1 Appendix). This effect was not significant for VD, the dementia subtype for which the present study had least power.

One strength of this study is that we examined subtypes of dementia, not only focusing on AD, for which the biological plausibility of a lithium effect is perhaps strongest, but also including others such as VD, for which corresponding evidence is more limited. Another advantage of our study is that we examined our results by the duration of lithium treatment.

One limitation is the handling of BPAD, which is a significant risk factor for dementia [4,38]. Lithium is primarily used for BPAD, but also as augmentation therapy for recurrent unipolar depression, another condition associated with increased risk of developing dementia. Some people have both diagnoses (and thus the sum of patient numbers with BPAD and depression is greater than the total number of people using lithium in the exposed cohort). To test the robustness of our results, we analysed (a) using coded BPAD as a covariate (which may underestimate the prevalence of BPAD because of diagnostic undercoding); and (b) assuming that the use of lithium itself implied the presence of BPAD (which may overestimate the prevalence of BPAD because lithium has other indications). The association of lithium with a reduction in incident dementia was seen in both analyses, but any underestimation of BPAD prevalence may have underestimated any protective functions of lithium. We conducted a further sensitivity analysis by excluding everyone diagnosed with recurrent depression, and our results remained consistent.

Another limitation is that our sample size of patients using lithium was smaller than other studies conducted in Denmark [18,19] and Taiwan [14]. This limitation was specifically reflected by the wide CIs for results relating to some durations of lithium exposure, although, again, sensitivity analyses remained consistent with our primary findings.

It is possible that there may have been selection bias if clinicians avoided prescribing lithium to patients with possible dementia. In previous research [15], however, the decision to prescribe lithium was independent of the cognitive status of patients. In addition, we excluded patients with a diagnosis of MCI made before, or <1 year after, starting lithium, and we adjusted our model (via IPW) to control further for baseline differences between lithium-exposed and nonexposed groups.

Finally, mental disorders (including dementias) and physical comorbidities may have been undercoded. This possibility always exists with the use of routinely collected clinical data and is an unmeasured potential source of power reduction and/or bias.

The main unanswered question from this work is the dose–response association between lithium within its therapeutic range and the incidence of dementia. The clinical context means that lithium levels primarily lay within its therapeutic range of 0.4 to 1.0 mmol/L (mean 0.61 mM, interquartile range 0.49 to 0.80 mM, as above), but at times, lithium levels in some patients may have been >1.0 mmol/L, with resultant potential for neurotoxicity, or subtherapeutic, either might alter the estimate of the protective effects of lithium, and the optimal level for any such protective effect is unknown. The nature of our data set, with incomplete lithium level data, did not allow us to explore the relationship between lithium levels and outcomes.

Caution should be exercised when drawing conclusions from the current study with regard to the general population. Our cohort differs from the general population, as our database was of patients treated for MH conditions, although the age structure of our database was similar to that of England. The frequency of dementia in our control cohort was higher than in the general population [39], as would be expected for a MH service. We cannot exclude the possibility that any lithium effect would be of a different magnitude in the general population. One study from Denmark [18] found that continued lithium treatment (lithium prescribed at least 20 times) was associated with a reduced rate of developing dementia in patients with bipolar disorder, reduced to the same level as the rate for the general population, but also that short-term lithium treatment (prescribed lithium 2 to 19 times) was associated with a lower rate of developing dementia than the general population. In addition, another study from the same Denmark team found that high lithium exposure in drinking water was associated with a lower incidence of dementia [17]. More studies involving the general population and lithium exposure are needed.

In conclusion, prescription lithium use was associated with a decreased incidence of dementia and its subtypes, including AD and VD, after accounting for confounding variables. This finding was robust to sensitivity analyses, with evidence for duration-dependent effects. This adds to the growing preclinical, epidemiological, and trial evidence that lithium may be a disease-modifying agent in dementia. The present study adds to this evidence base in people with preexisting mental disorders, primarily BPAD. Since lithium is already established as a treatment of choice for BPAD [40], large-scale dose/effect observational studies, studies involving the general population, and studies focusing on other dementias (such as Lewy body dementia and Parkinson disease dementia) are now required, as well as definitive large-scale randomised trials of lithium for the prevention of progression to dementia in those with MCI or early disease.

## Supporting information

S1 Checklist STROBE checklist STROBE, Strengthening the Reporting of Observational Studies in Epidemiology.
(DOCX)

S1 Appendix Additional information on study methods and findings. **Fig A:** Association of lithium use with the development of dementia and its subtypes by Cox proportional hazards models: sensitivity analysis with a longer (2-year) criterion for identifying medications/comorbidities (see Methods). Adjusted HRs, 95% CIs, and *p*-values were extracted from inverse probability weighted Cox regression. Adjusted for age, sex, marital status, ethnicity, smoking status, alcohol disorders, antipsychotic use, depression, mania or BPAD, hypertension, central vascular disease, diabetes mellitus, and hyperlipidemias. **Fig B:** Association of duration of lithium exposure with the development of dementia and its subtypes by Cox proportional hazards models: sensitivity analysis with a longer (2-year) criterion for identifying medications/comorbidities (see Methods). Adjusted HRs, 95% CIs, and *p*-values were extracted from inverse probability weighted Cox regression. Adjusted for age, sex, marital status, ethnicity, smoking status, alcohol disorders, antipsychotic use, depression, mania or BPAD, hypertension, central vascular disease, diabetes mellitus, and hyperlipidemias. NA indicates no result (no corresponding cases). **Fig C:** Association of lithium use with the development of dementia and its subtypes by Cox proportional hazards models: sensitivity analysis requiring at least 2 years of follow-up. Adjusted HRs, 95% CIs, and *p*-values were extracted from inverse probability weighted Cox regression. Adjusted for age, sex, marital status, ethnicity, smoking status, alcohol disorders, antipsychotic use, depression, mania or BPAD, hypertension, central vascular disease, diabetes mellitus, and hyperlipidemias. **Fig D:** Association of duration of lithium exposure with the development of dementia and its subtypes by Cox proportional hazards models: sensitivity analysis requiring at least 2 years of follow-up. Adjusted HRs, 95% CIs, and *p*-values were extracted from inverse probability weighted Cox regression. Adjusted for age, sex, marital status, ethnicity, smoking status, alcohol disorders, antipsychotic use, depression, mania or BPAD, hypertension, central vascular disease, diabetes mellitus, and hyperlipidemias. NA indicates no result (no corresponding cases). **Fig E:** Association of lithium with the development of dementia and its subtypes by Cox proportional hazards models: sensitivity analysis assuming that all lithium users had BPAD. Adjusted HRs, 95% CIs, and *p*-values were extracted from inverse probability weighted Cox regression. Adjusted for age, sex, marital status, ethnicity, smoking status, alcohol disorders, antipsychotic use, depression, mania or BPAD, hypertension, central vascular disease, diabetes mellitus, and hyperlipidemias. **Fig F:** Association of duration of lithium exposure with the development of dementia and its subtypes by Cox proportional hazards models: sensitivity analysis assuming that all lithium users had BPAD. Adjusted HRs, 95% CIs, and *p*-values were extracted from inverse probability weighted Cox regression. Adjusted for age, sex, marital status, ethnicity, smoking status, alcohol disorders, antipsychotic use, depression, mania or BPAD, hypertension, central vascular disease, diabetes mellitus, and hyperlipidemias. NA indicates no result (no corresponding cases). **Fig G:** Association of lithium with the development of dementia and its subtypes by Cox proportional hazards models: sensitivity analysis by excluding people diagnosed with recurrent depression. Adjusted HRs, 95% CIs, and *p*-values were extracted from inverse probability weighted Cox regression. Adjusted for age, sex, marital status, ethnicity, smoking status, alcohol disorders, antipsychotic use, depression, mania or BPAD, hypertension, central vascular disease, diabetes mellitus, and hyperlipidemias. **Fig H:** Association of duration of lithium exposure with the development of dementia and its subtypes by Cox proportional hazards models: sensitivity analysis by excluding people diagnosed with recurrent depression. Adjusted HRs, 95% CIs, and *p*-values were extracted from inverse probability weighted Cox regression. Adjusted for age, sex, marital status, ethnicity, smoking status, alcohol disorders, antipsychotic use, depression, mania or BPAD, hypertension, central vascular disease, diabetes mellitus, and hyperlipidemias. NA indicates no result (no corresponding cases). **Fig I:** Association of lithium with the development of dementia and its subtypes by Cox proportional hazards models: sensitivity analysis by excluding the ethnicity variable. Adjusted HRs, 95% CIs, and *p*-values were extracted from inverse probability weighted Cox regression. Adjusted for age, sex, marital status, smoking status, alcohol disorders, antipsychotic use, depression, mania or BPAD, hypertension, central vascular disease, diabetes mellitus, and hyperlipidemias. **Fig J:** Association of duration of lithium exposure with the development of dementia and its subtypes by Cox proportional hazards models: sensitivity analysis by excluding the ethnicity variable. Adjusted HRs, 95% CIs, and *p*-values were extracted from inverse probability weighted Cox regression. Adjusted for age, sex, marital status, smoking status, alcohol disorders, antipsychotic use, depression, mania or BPAD, hypertension, central vascular disease, diabetes mellitus, and hyperlipidemias. **Fig K:** Association of lithium with the development of dementia and its subtypes by Cox proportional hazards models: sensitivity analysis by considering competing effects from death. Adjusted HRs, 95% CIs, and *p*-values were extracted from inverse probability weighted Cox regression. Adjusted for age, sex, marital status, ethnicity, smoking status, alcohol disorders, antipsychotic use, depression, mania or BPAD, hypertension, central vascular disease, diabetes mellitus, and hyperlipidemias. **Fig L:** Association of duration of lithium exposure with the development of dementia and its subtypes by Cox proportional hazards models: sensitivity analysis by considering competing effects from death. Adjusted HRs, 95% CIs, and *p*-values were extracted from inverse probability weighted Cox regression. Adjusted for age, sex, marital status, ethnicity, smoking status, alcohol disorders, antipsychotic use, depression, mania or BPAD, hypertension, central vascular disease, diabetes mellitus, and hyperlipidemias. NA indicates no result (no corresponding cases). **Fig M:** Association of lithium with the development of dementia and its subtypes by Cox proportional hazards models: sensitivity analysis by only controlling for confounders known at baseline. Adjusted HRs, 95% CIs, and *p*-values were extracted from inverse probability weighted Cox regression. Adjusted for age, sex, marital status, ethnicity, smoking status, alcohol disorders, antipsychotic use, depression, mania or BPAD, hypertension, central vascular disease, diabetes mellitus, and hyperlipidemias. **Fig N:** Association of duration of lithium exposure with the development of dementia and its subtypes by Cox proportional hazards models: sensitivity analysis by only controlling for confounders known at baseline. Adjusted HRs, 95% CIs, and *p*-values were extracted from inverse probability weighted Cox regression. Adjusted for age, sex, marital status, ethnicity, smoking status, alcohol disorders, antipsychotic use, depression, mania or BPAD, hypertension, central vascular disease, diabetes mellitus, and hyperlipidemias. NA indicates no result (no corresponding cases). **Fig O:** Association of lithium with the development of dementia and its subtypes by Cox proportional hazards models: sensitivity analysis by treating exposure to lithium as a time-varying variable. Adjusted HRs, 95% CIs, and *p*-values were extracted from inverse probability weighted Cox regression. Adjusted for age, sex, marital status, ethnicity, smoking status, alcohol disorders, antipsychotic use, depression, mania or BPAD, hypertension, central vascular disease, diabetes mellitus, and hyperlipidemias. **Fig P:** Association of duration of lithium exposure with the development of dementia and its subtypes by Cox proportional hazards models: sensitivity analysis by treating the duration of exposure to lithium as a time-varying variable. Adjusted HRs, 95% CIs, and *p*-values were extracted from inverse probability weighted Cox regression. Adjusted for age, sex, marital status, ethnicity, smoking status, alcohol disorders, antipsychotic use, depression, mania or BPAD, hypertension, central vascular disease, diabetes mellitus, and hyperlipidemias. **Fig Q:** Association of duration of lithium exposure with the development of dementia and its subtypes by Cox proportional hazards models: sensitivity analysis by pooling intermediate lithium exposure durations. Adjusted HRs, 95% CIs, and *p*-values were extracted from inverse probability weighted Cox regression. Adjusted for age, sex, marital status, ethnicity, smoking status, alcohol disorders, antipsychotic use, depression, mania or BPAD, hypertension, central vascular disease, diabetes mellitus, and hyperlipidemias. **Fig R:** Association of duration of lithium exposure with the development of dementia and its subtypes by Cox proportional hazards models: sensitivity analysis by adding an interaction term between lithium exposure and exposure duration (in addition to the main effect of lithium exposure). HRs are for exposure duration (the interaction term); thus, HRs < 1 indicate a progressive reduction in the risk of dementia for each additional year of lithium exposure (over and above the fact of lithium exposure). Unadjusted/adjusted HRs, 95% CIs, and *p*-values were extracted from inverse probability weighted Cox regression. Adjusted for age, sex, marital status, ethnicity, smoking status, alcohol disorders, antipsychotic use, depression, mania or BPAD, hypertension, central vascular disease, diabetes mellitus, and hyperlipidemias. BPAD, bipolar affective disorder/mania; CI, confidence interval; HR, hazard ratio.
(DOCX)

## Abbreviations

AD: Alzheimer disease
AIC: Akaike information criterion
BPAD: bipolar affective disorder/mania
CI: confidence interval
CPFT: Cambridgeshire and Peterborough NHS Foundation Trust
HR: hazard ratio
ICD-10: International Classification of Diseases-10th Revision
IPW: inverse probability weights/weighting
MCI: mild cognitive impairment
MH: mental health
NICE: National Institute for Health and Care Excellence
NLP: natural language processing
RCT: randomised controlled trial
SMD: standardised mean difference
STROBE: Strengthening the Reporting of Observational Studies in Epidemiology
VD: vascular dementia
WHO: World Health Organization
