# Supplementary material for: Association between lithium use and the incidence of dementia and its subtypes: A retrospective cohort study
Source: PLoS Med. 2022 Mar 17;19(3):e1003941. doi: 10.1371/journal.pmed.1003941 (PMC8929585; doi:10.1371/journal.pmed.1003941)

**Table A: List of medicines**

| Antipsychotics | **First-generation:** Benperidol, chlorpromazine, flupentixol, fluphenazine, haloperidol, levomepromazine, pericyazine, perphenazine, pimozide, pipotiazine, prochlorperazine, promazine, trifluoperazine, zuclopenthixol  **Second-generation:** Asenapine, amisulpride, aripiprazole, clozapine, iloperidone, lurasidone, olanzapine, paliperidone, quetiapine, risperidone, sertindole, sulpiride, ziprasidone, zotepine |
| --- | --- |
| Antidepressants | Agomelatine, amitriptyline, bupropion, citalopram, clomipramine, dosulepin, doxepin, duloxetine, escitalopram, fluoxetine, fluvoxamine, imipramine, isocarboxazid, lofepramine, maprotiline, mianserin, mirtazapine, moclobemide, nefazodone, nortriptyline, paroxetine, phenelzine, reboxetine, sertraline, tranylcypromine, trazodone, trimipramine, tryptophan, venlafaxine, vortioxetine |
| Hypoglycaemic agents | Acarbose, alogliptin, canagliflozin, dapagliflozin, empagliflozin, exenatide, glibenclamide, gliclazide, glimepiride, glipizide, insulin, linagliptin, liraglutide, lixisenatide, metformin, nateglinide, pioglitazone, repaglinide, saxagliptin, sitagliptin, tolbutamide, vildagliptin |
| ACE inhibitors | Captopril, enalapril, fosinopril, imidapril, lisinopril, perindopril, quinapril, ramipril, trandolapril |
| Angiotensin-II receptor antagonists | Azilsartan, candesartan, eprosartan, irbesartan, losartan, olmesartan, telmisartan, valsartan |
| Beta blockers | Acebutolol, atenolol, bisoprolol, carvedilol, celiprolol hydrochloride  Labetalol, metoprolol, nadolol, nebivolol, pindolol, sotalol |
| Calcium channel antagonists | Amlodipine, diltiazem, elodipine, felodipine, lacidipine, lercanidipine, nicardipine, nifedipine, nimodipine, verapamil |
| Diuretics | **Thiazides:**  Bendroflumethiazide, chlortalidone, indapamide, metolazone, xipamide  **Loop diuretics:**  Bumetanide, furosemide, torasemide  **Potassium-sparing diuretics:**  Amiloride, triamterene  **Aldosterone antagonists:**  Eplerenone, spironolactone |
| Lipid-lowering medication | **Statins:**  Atorvastatin, fluvastatin, pravastatin, rosuvastatin, simvastatin  **Others:**  Colestyramine, colestipol, ezetimibe, fenofibrate |
| Corticosteroids, bronchodilators and other anti-inflammatory drugs for airways disease | **Corticosteroids:**  Alclometasone, betamethasone, clobetasol, clobetasone butyrate, fluocortolone, dexamethasone, fludrocortisone, fludroxycortide, flumetasone, fluocinolone, fluocinonide, fluorometholone, hydrocortisone, loteprednol, methylprednisolone, mometasone, prednisolone, triamcinolone  **Inhaled corticosteroids:**  Beclomethasone, budesonide, ciclesonide, fluticasone  **Bronchodilators and anti-inflammatory drugs used for airways disease:**  Aminophylline, formoterol, ipratropium, mepolizumab, montelukast, nedocromil sodium, omalizumab, reslizumab, salbutamol, salmeterol, sodium cromoglicate, terbutaline, theophylline, tiotropium |

**Fig A. Association of lithium use with the development of dementia and its subtypes by Cox proportional hazards models: sensitivity analysis with a longer (2-year) criterion for identifying medications/comorbidities (see Methods).** Adjusted hazard ratios (HRs), 95% confidence intervals (CI), and p values were extracted from inverse-probability-weighted Cox regression. Adjusted for age, sex, marital status, ethnicity, smoking status, alcohol disorders, antipsychotic use, depression, mania or bipolar affective disorder, hypertension, central vascular disease, diabetes mellitus, and hyperlipidaemias.


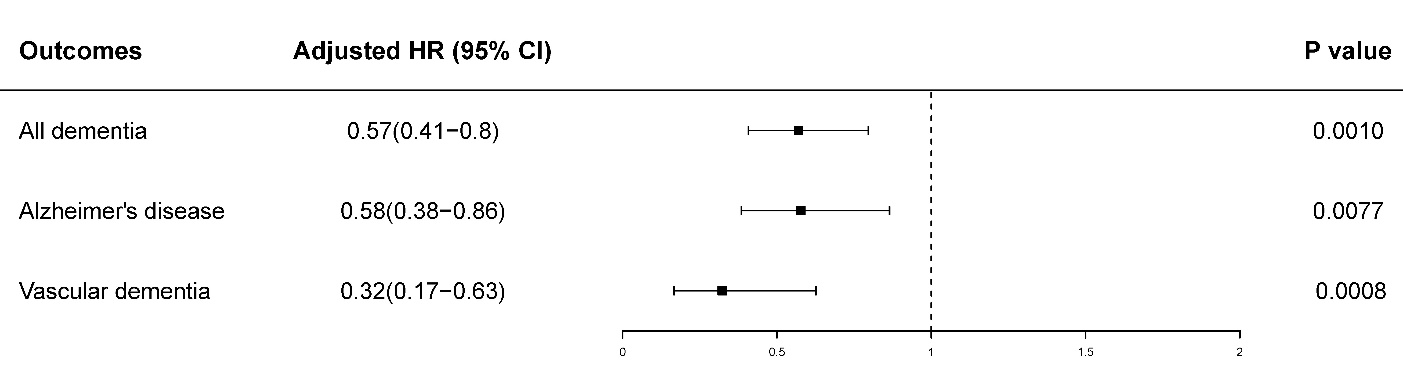


**Fig B. Association of duration of lithium exposure with the development of dementia and its subtypes by Cox proportional hazards models: sensitivity analysis with a longer (2-year) criterion for identifying medications/comorbidities (see Methods).** Adjusted hazard ratios (HRs), 95% confidence intervals (CI), and p values were extracted from inverse-probability-weighted Cox regression. Adjusted for age, sex, marital status, ethnicity, smoking status, alcohol disorders, antipsychotic use, depression, mania or bipolar affective disorder, hypertension, central vascular disease, diabetes mellitus, and hyperlipidaemias. NA indicates no result (no corresponding cases).


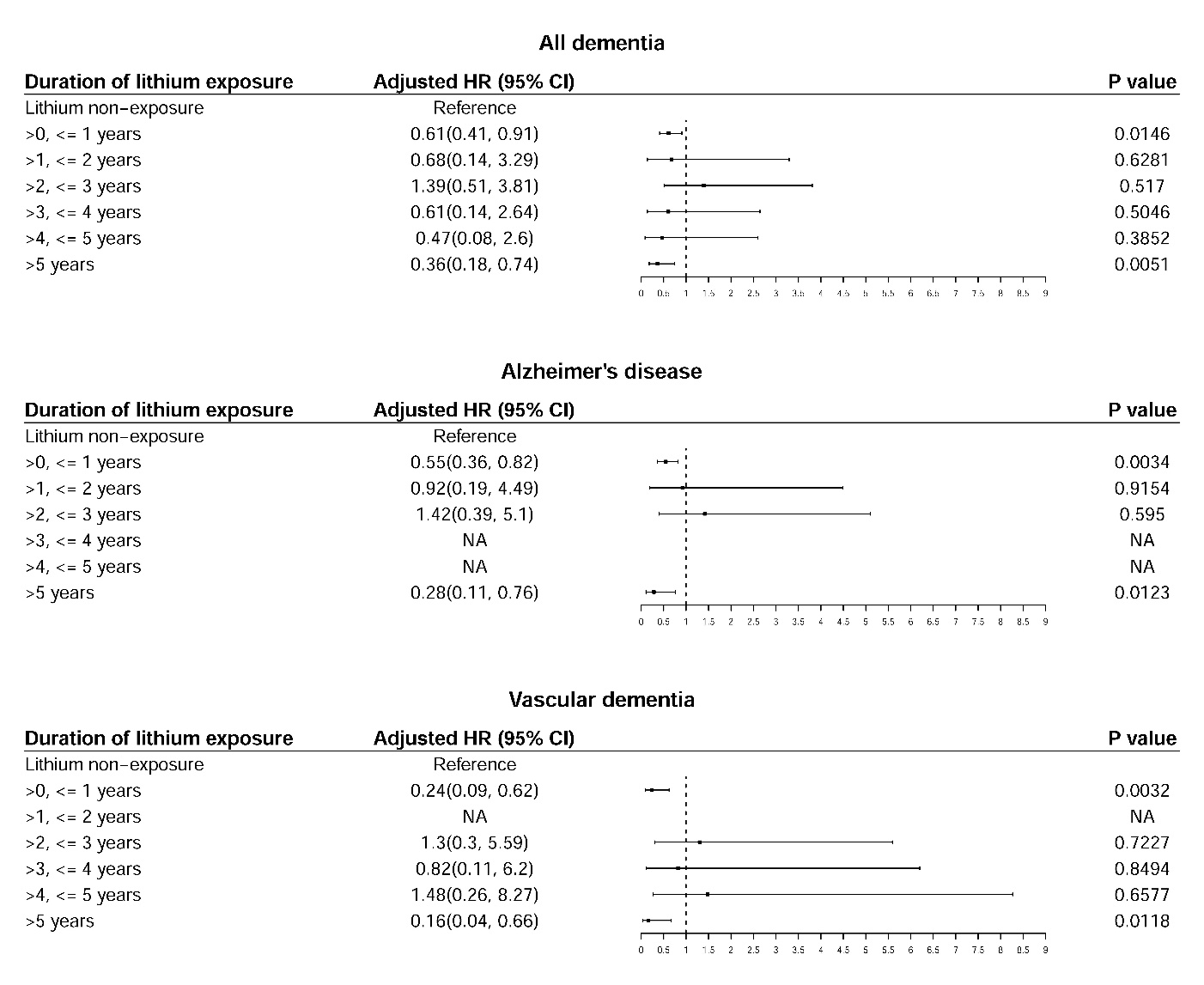


**Fig C. Association of lithium use with the development of dementia and its subtypes by Cox proportional hazards models: sensitivity analysis requiring at least two years of follow-up.** Adjusted hazard ratios (HRs), 95% confidence intervals (CI), and p values were extracted from inverse-probability-weighted Cox regression. Adjusted for age, sex, marital status, ethnicity, smoking status, alcohol disorders, antipsychotic use, depression, mania or bipolar affective disorder, hypertension, central vascular disease, diabetes mellitus, and hyperlipidaemias.


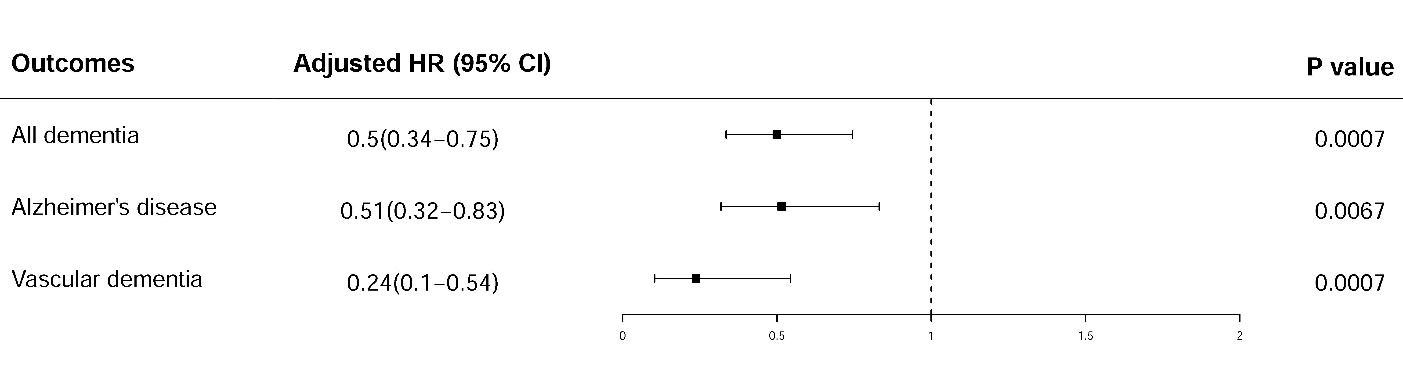


**Fig D. Association of duration of lithium exposure with the development of dementia and its subtypes by Cox proportional hazards models: sensitivity analysis requiring at least two years of follow-up.** Adjusted hazard ratios (HRs), 95% confidence intervals (CI), and p values were extracted from inverse-probability-weighted Cox regression. Adjusted for age, sex, marital status, ethnicity, smoking status, alcohol disorders, antipsychotic use, depression, mania or bipolar affective disorder, hypertension, central vascular disease, diabetes mellitus, and hyperlipidaemias. NA indicates no result (no corresponding cases).


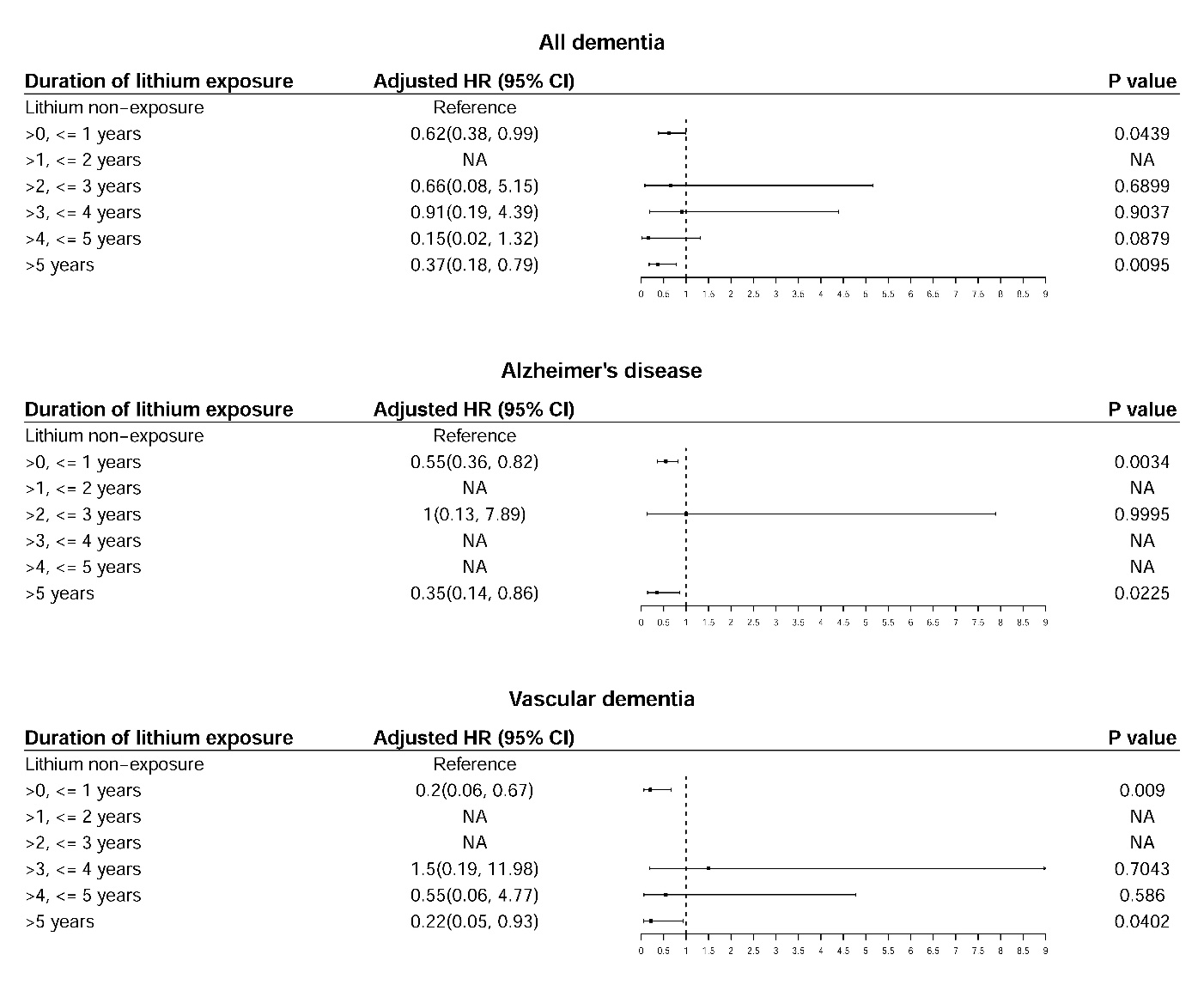


**Fig E. Association of lithium with the development of dementia and its subtypes by Cox proportional hazards models: sensitivity analysis assuming that all lithium users had bipolar affective disorder.** Adjusted hazard ratios (HRs), 95% confidence intervals (CI), and p values were extracted from inverse-probability-weighted Cox regression. Adjusted for age, sex, marital status, ethnicity, smoking status, alcohol disorders, antipsychotic use, depression, mania or bipolar affective disorder, hypertension, central vascular disease, diabetes mellitus, and hyperlipidaemias.


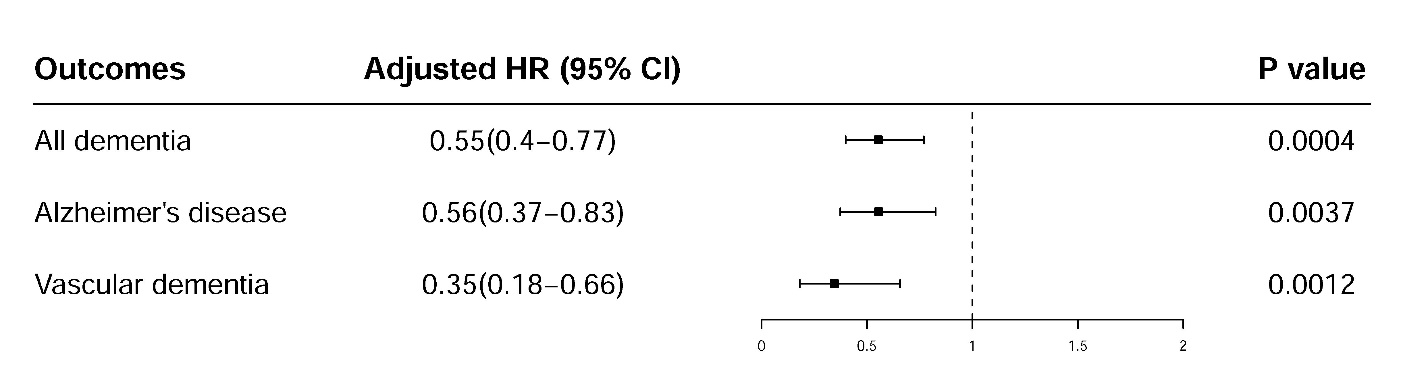


**Fig F. Association of duration of lithium exposure with the development of dementia and its subtypes by Cox proportional hazards models: sensitivity analysis assuming that all lithium users had bipolar affective disorder.** Adjusted hazard ratios (HRs), 95% confidence intervals (CI), and p values were extracted from inverse-probability-weighted Cox regression. Adjusted for age, sex, marital status, ethnicity, smoking status, alcohol disorders, antipsychotic use, depression, mania or bipolar affective disorder, hypertension, central vascular disease, diabetes mellitus, and hyperlipidaemias. NA indicates no result (no corresponding cases).


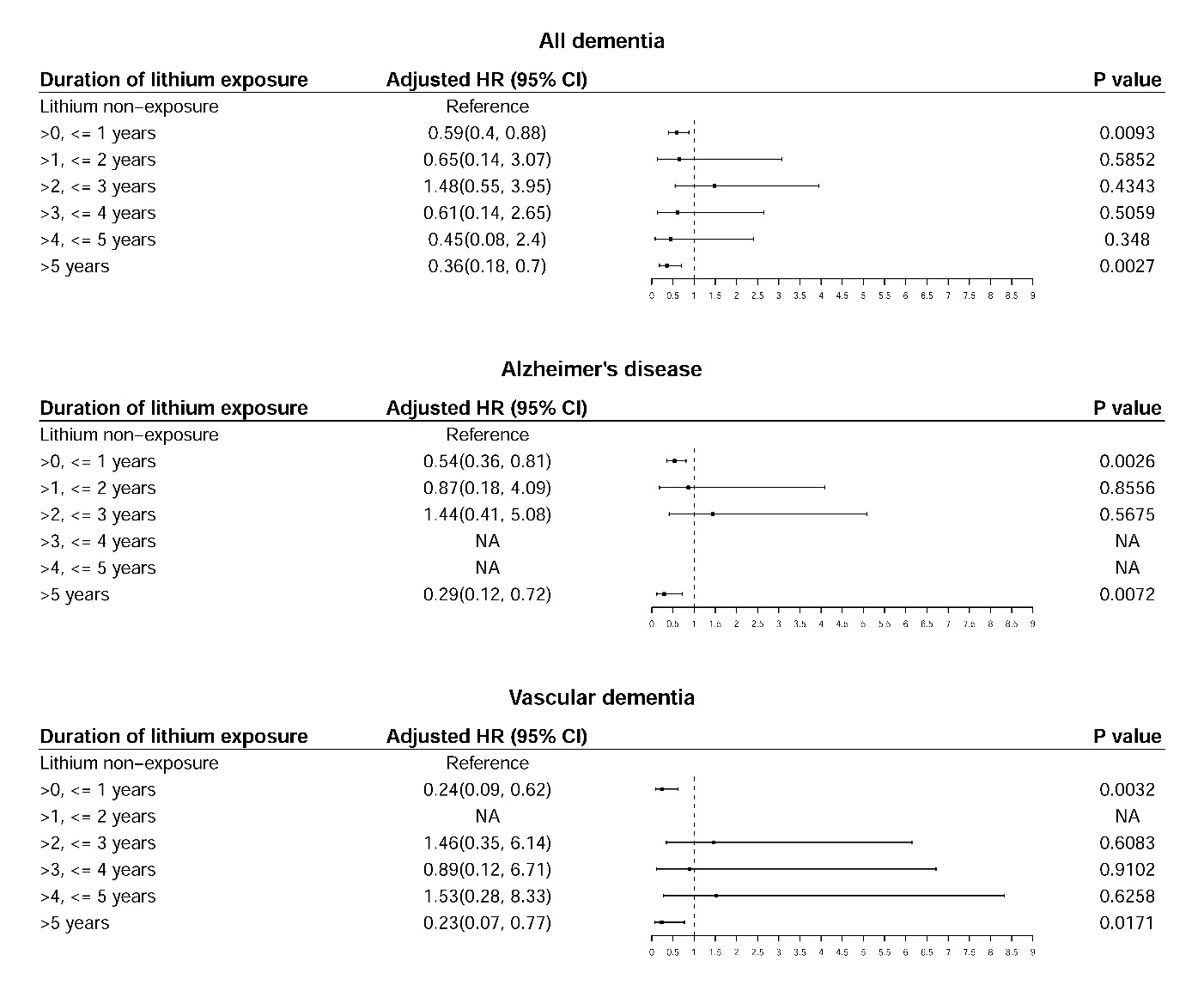


**Fig G. Association of lithium with the development of dementia and its subtypes by Cox proportional hazards models: sensitivity analysis by excluding people diagnosed with recurrent depression.** Adjusted hazard ratios (HRs), 95% confidence intervals (CI), and p values were extracted from inverse-probability-weighted Cox regression. Adjusted for age, sex, marital status, ethnicity, smoking status, alcohol disorders, antipsychotic use, depression, mania or bipolar affective disorder, hypertension, central vascular disease, diabetes mellitus, and hyperlipidaemias.


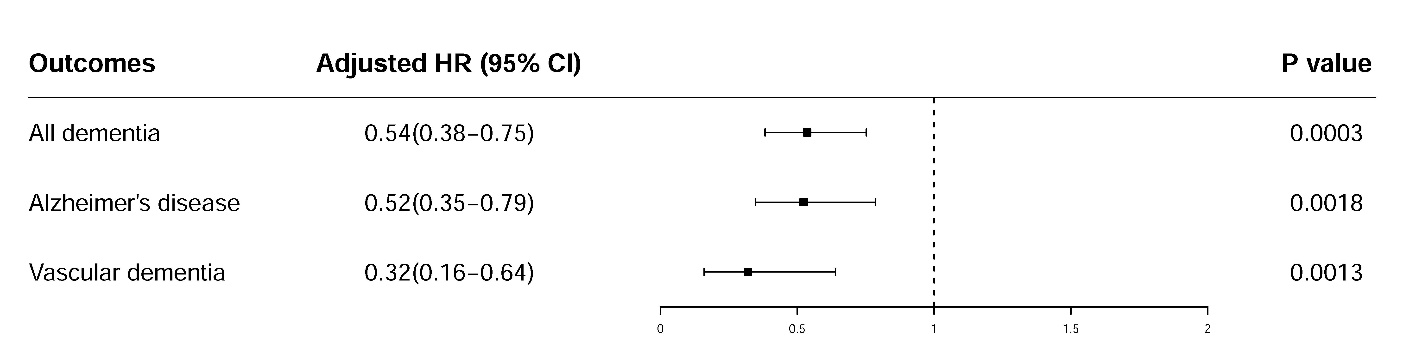


**Fig H. Association of duration of lithium exposure with the development of dementia and its subtypes by Cox proportional hazards models: sensitivity analysis by excluding people diagnosed with recurrent depression.** Adjusted hazard ratios (HRs), 95% confidence intervals (CI), and p values were extracted from inverse-probability-weighted Cox regression. Adjusted for age, sex, marital status, ethnicity, smoking status, alcohol disorders, antipsychotic use, depression, mania or bipolar affective disorder, hypertension, central vascular disease, diabetes mellitus, and hyperlipidaemias. NA indicates no result (no corresponding cases).


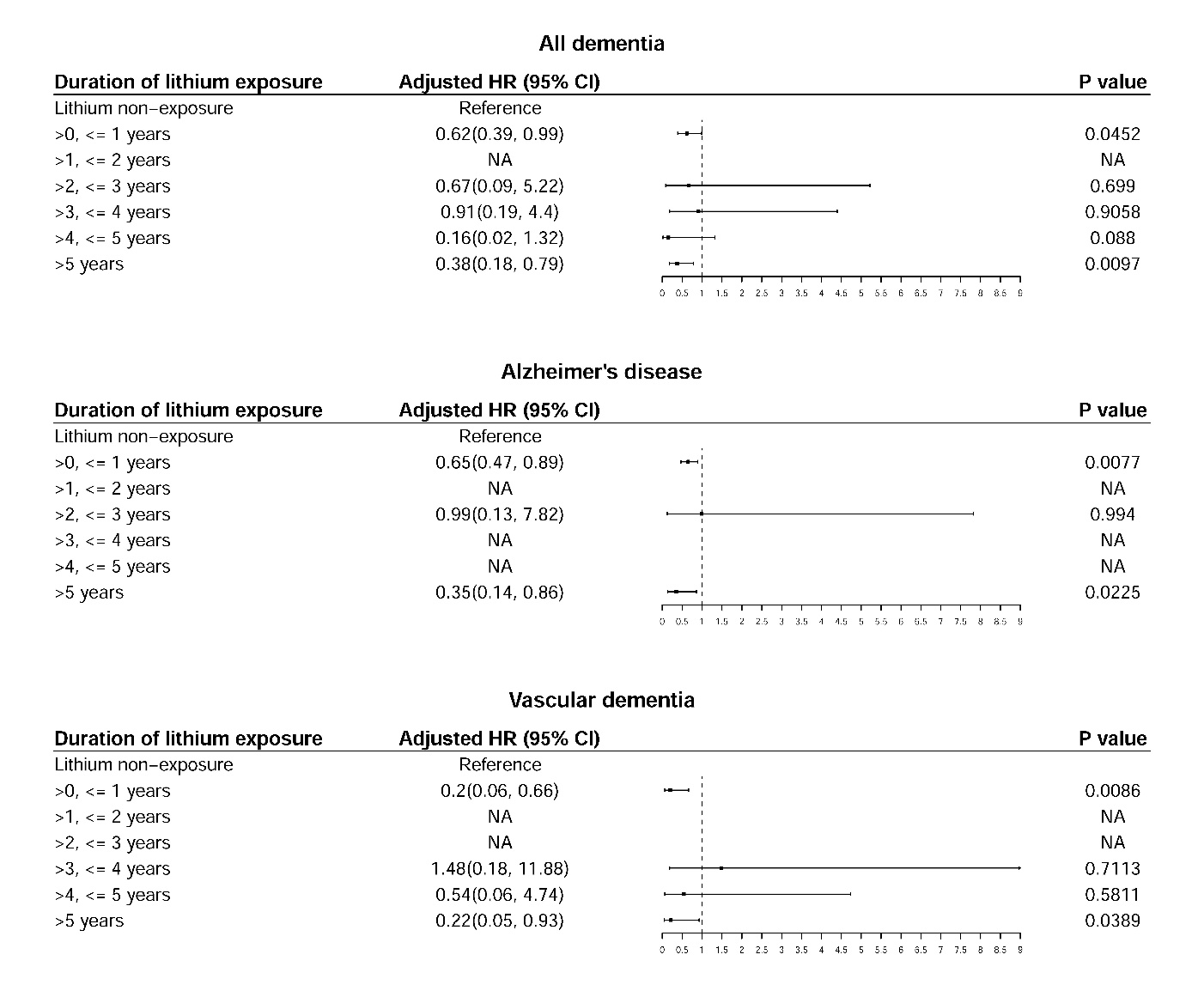


**Fig I. Association of lithium with the development of dementia and its subtypes by Cox proportional hazards models: sensitivity analysis by excluding the ethnicity variable.** Adjusted hazard ratios (HRs), 95% confidence intervals (CI), and p values were extracted from inverse-probability-weighted Cox regression. Adjusted for age, sex, marital status, smoking status, alcohol disorders, antipsychotic use, depression, mania or bipolar affective disorder, hypertension, central vascular disease, diabetes mellitus, and hyperlipidaemias.


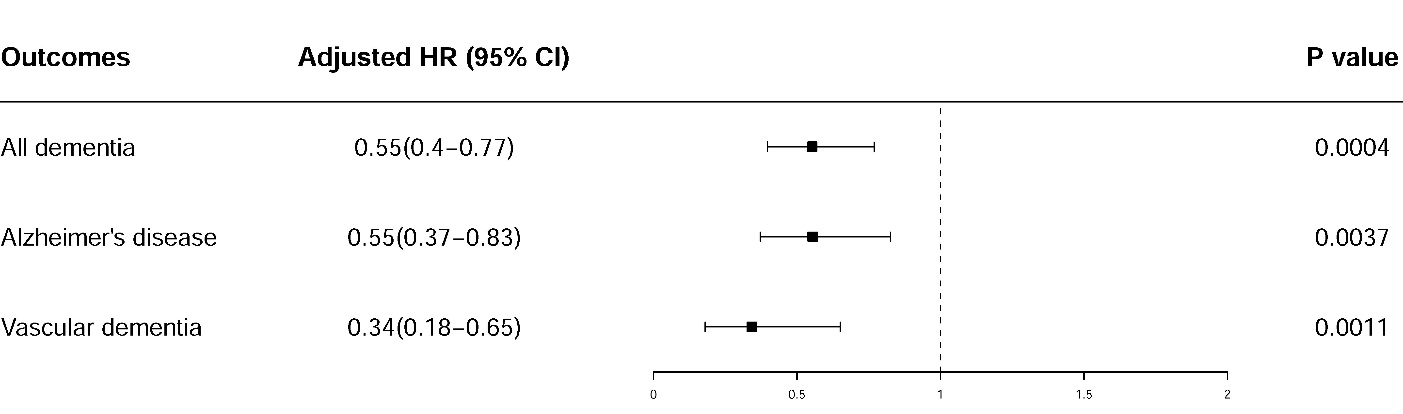


**Fig J. Association of duration of lithium exposure with the development of dementia and its subtypes by Cox proportional hazards models: sensitivity analysis by excluding the ethnicity variable.** Adjusted hazard ratios (HRs), 95% confidence intervals (CI), and p values were extracted from inverse-probability-weighted Cox regression. Adjusted for age, sex, marital status, smoking status, alcohol disorders, antipsychotic use, depression, mania or bipolar affective disorder, hypertension, central vascular disease, diabetes mellitus, and hyperlipidaemias.


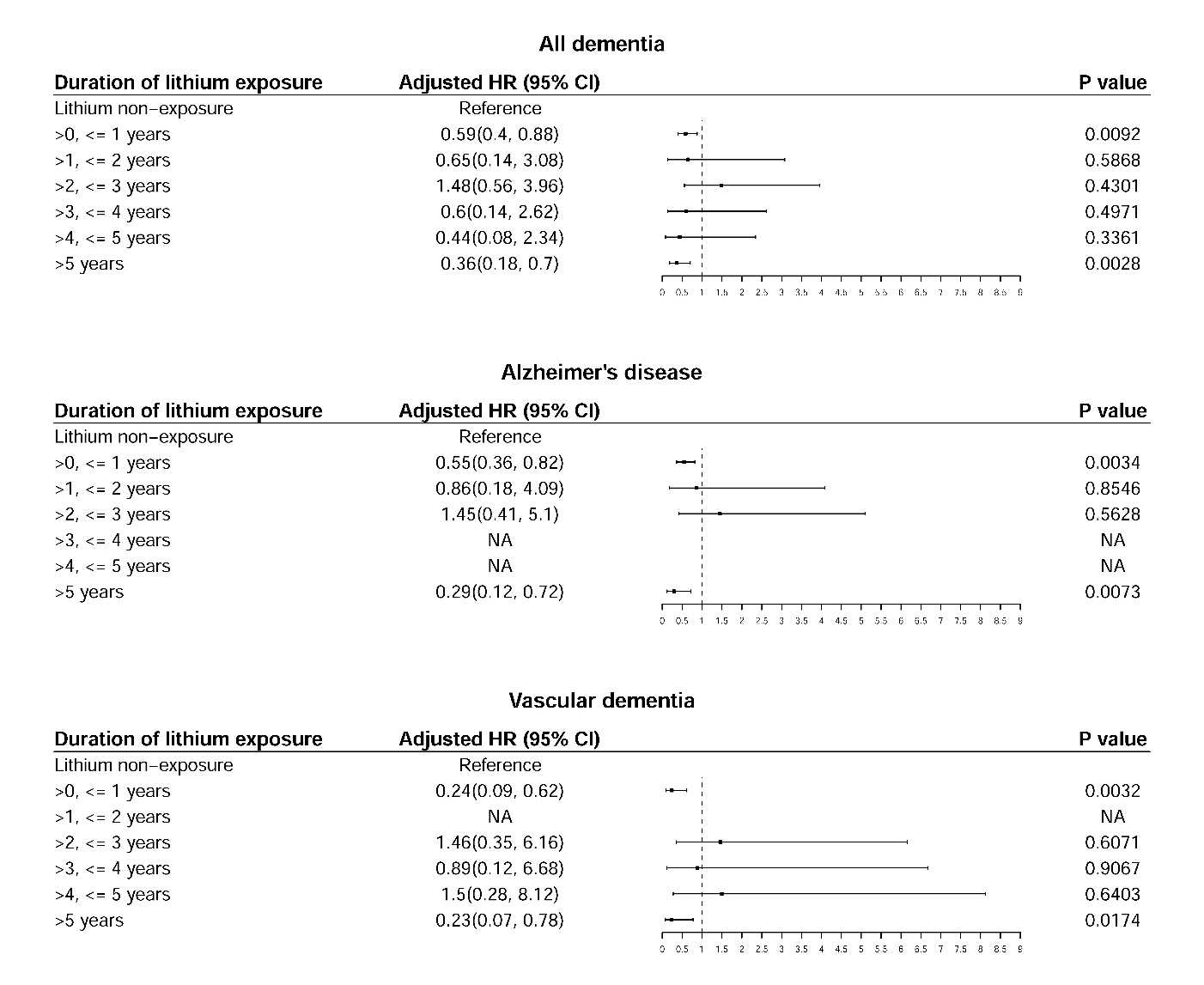


**Fig K. Association of lithium with the development of dementia and its subtypes by Cox proportional hazards models: sensitivity analysis by considering competing effects from death.** Adjusted hazard ratios (HRs), 95% confidence intervals (CI), and p values were extracted from inverse-probability-weighted Cox regression. Adjusted for age, sex, marital status, ethnicity, smoking status, alcohol disorders, antipsychotic use, depression, mania or bipolar affective disorder, hypertension, central vascular disease, diabetes mellitus, and hyperlipidaemias.


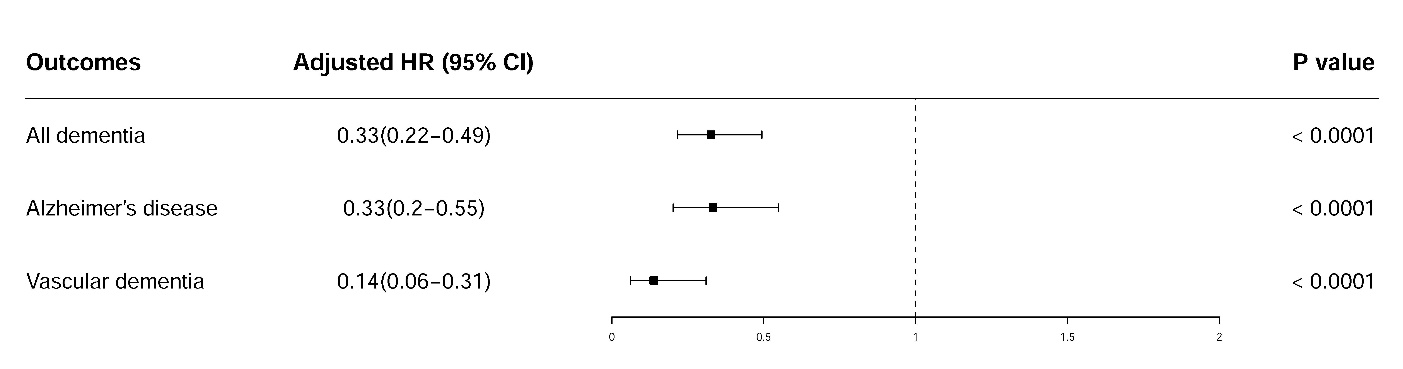


**Fig L. Association of duration of lithium exposure with the development of dementia and its subtypes by Cox proportional hazards models: sensitivity analysis by considering competing effects from death.** Adjusted hazard ratios (HRs), 95% confidence intervals (CI), and p values were extracted from inverse-probability-weighted Cox regression. Adjusted for age, sex, marital status, ethnicity, smoking status, alcohol disorders, antipsychotic use, depression, mania or bipolar affective disorder, hypertension, central vascular disease, diabetes mellitus, and hyperlipidaemias. NA indicates no result (no corresponding cases).


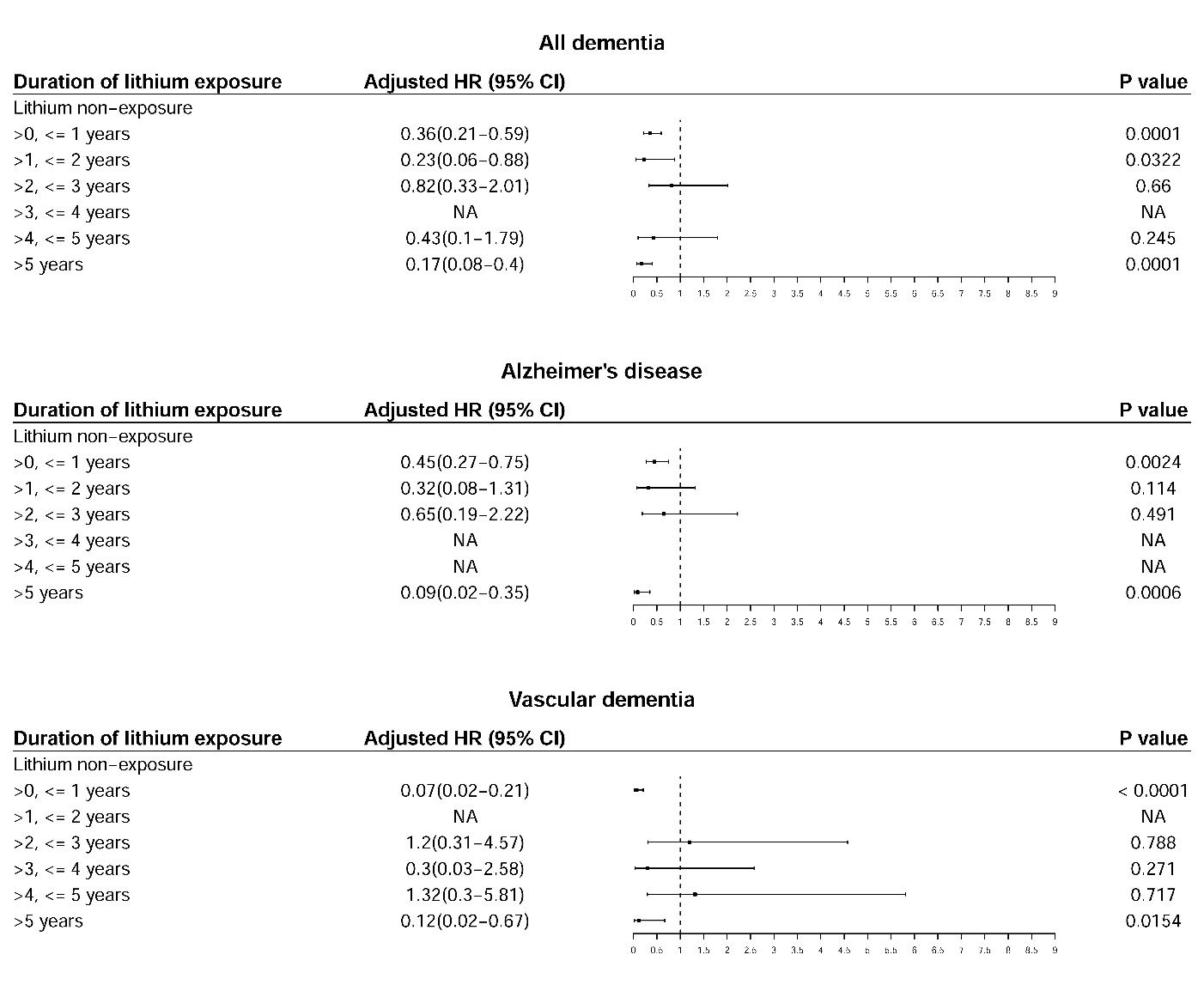


**Fig M. Association of lithium with the development of dementia and its subtypes by Cox proportional hazards models: sensitivity analysis by only controlling for confounders known at baseline.** Adjusted hazard ratios (HRs), 95% confidence intervals (CI), and p values were extracted from inverse-probability-weighted Cox regression. Adjusted for age, sex, marital status, ethnicity, smoking status, alcohol disorders, antipsychotic use, depression, mania or bipolar affective disorder, hypertension, central vascular disease, diabetes mellitus, and hyperlipidaemias.


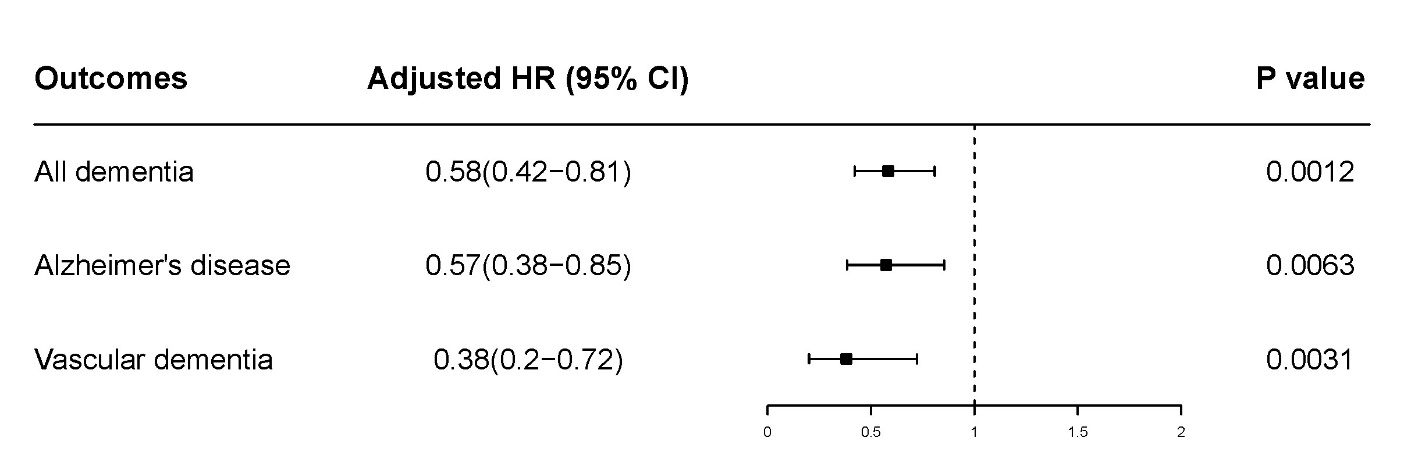


**Fig N. Association of duration of lithium exposure with the development of dementia and its subtypes by Cox proportional hazards models: sensitivity analysis by only controlling for confounders known at baseline.** Adjusted hazard ratios (HRs), 95% confidence intervals (CI), and p values were extracted from inverse-probability-weighted Cox regression. Adjusted for age, sex, marital status, ethnicity, smoking status, alcohol disorders, antipsychotic use, depression, mania or bipolar affective disorder, hypertension, central vascular disease, diabetes mellitus, and hyperlipidaemias. NA indicates no result (no corresponding cases).


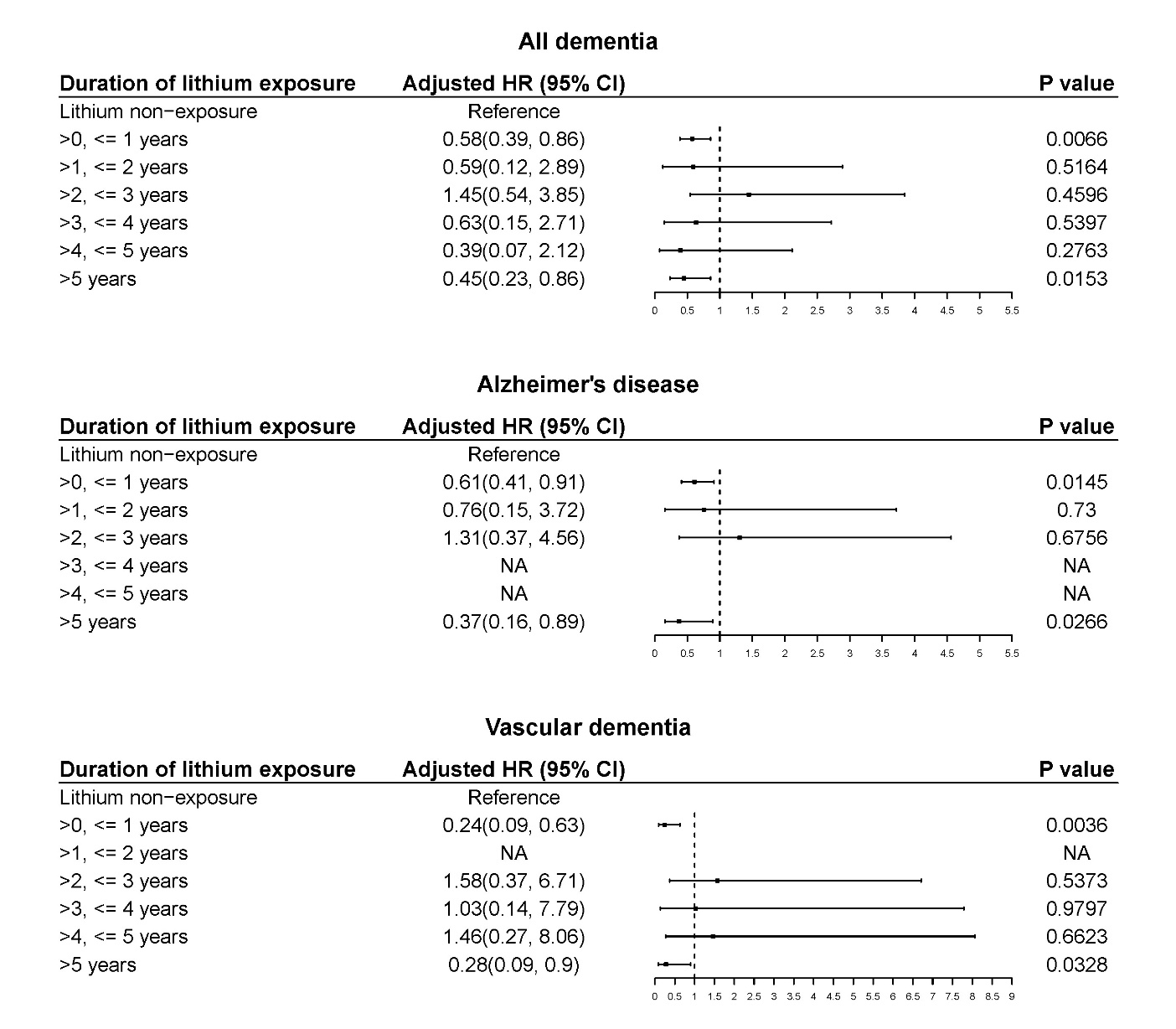


**Fig O. Association of lithium with the development of dementia and its subtypes by Cox proportional hazards models: sensitivity analysis by treating exposure to lithium as a time-varying variable.** Adjusted hazard ratios (HRs), 95% confidence intervals (CI), and p values were extracted from inverse-probability-weighted Cox regression. Adjusted for age, sex, marital status, ethnicity, smoking status, alcohol disorders, antipsychotic use, depression, mania or bipolar affective disorder, hypertension, central vascular disease, diabetes mellitus, and hyperlipidaemias.


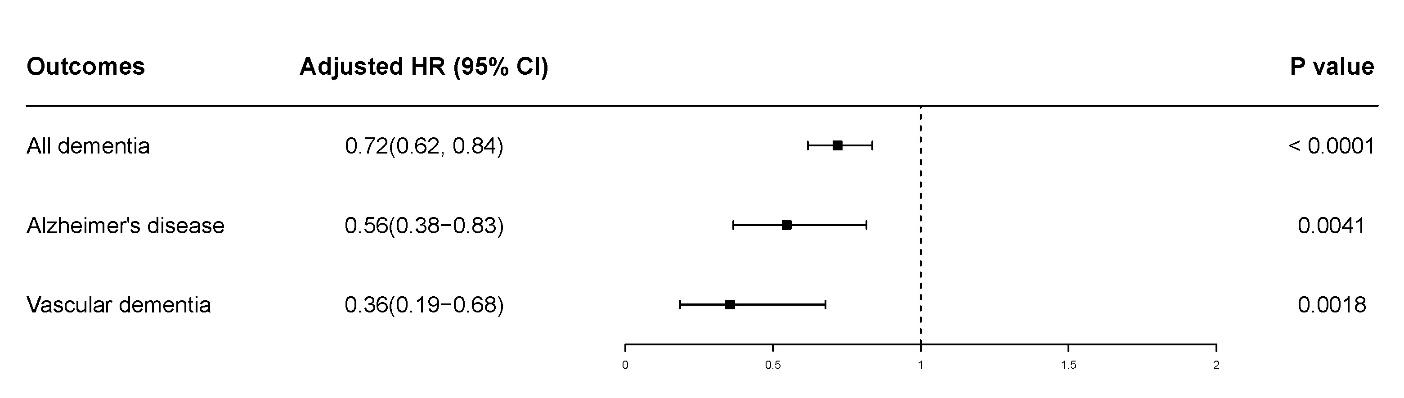


**Fig P. Association of duration of lithium exposure with the development of dementia and its subtypes by Cox proportional hazards models: sensitivity analysis by treating the duration of exposure to lithium as a time-varying variable.** Adjusted hazard ratios (HRs), 95% confidence intervals (CI), and p values were extracted from inverse-probability-weighted Cox regression. Adjusted for age, sex, marital status, ethnicity, smoking status, alcohol disorders, antipsychotic use, depression, mania or bipolar affective disorder, hypertension, central vascular disease, diabetes mellitus, and hyperlipidaemias.


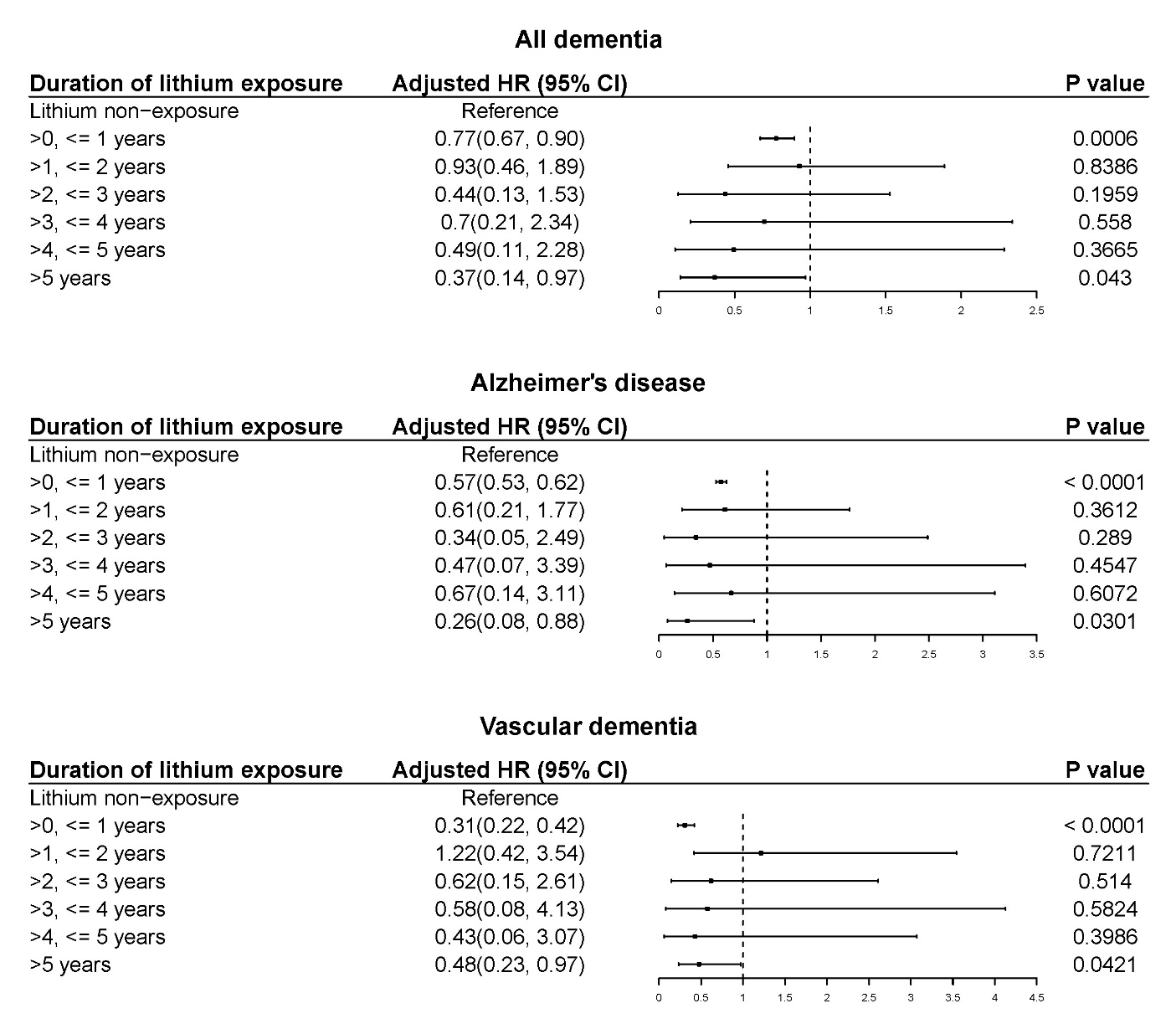


**Fig Q. Association of duration of lithium exposure with the development of dementia and its subtypes by Cox proportional hazards models: sensitivity analysis by pooling intermediate lithium exposure durations.** Adjusted hazard ratios (HRs), 95% confidence intervals (CI), and p values were extracted from inverse-probability-weighted Cox regression. Adjusted for age, sex, marital status, ethnicity, smoking status, alcohol disorders, antipsychotic use, depression, mania or bipolar affective disorder, hypertension, central vascular disease, diabetes mellitus, and hyperlipidaemias.


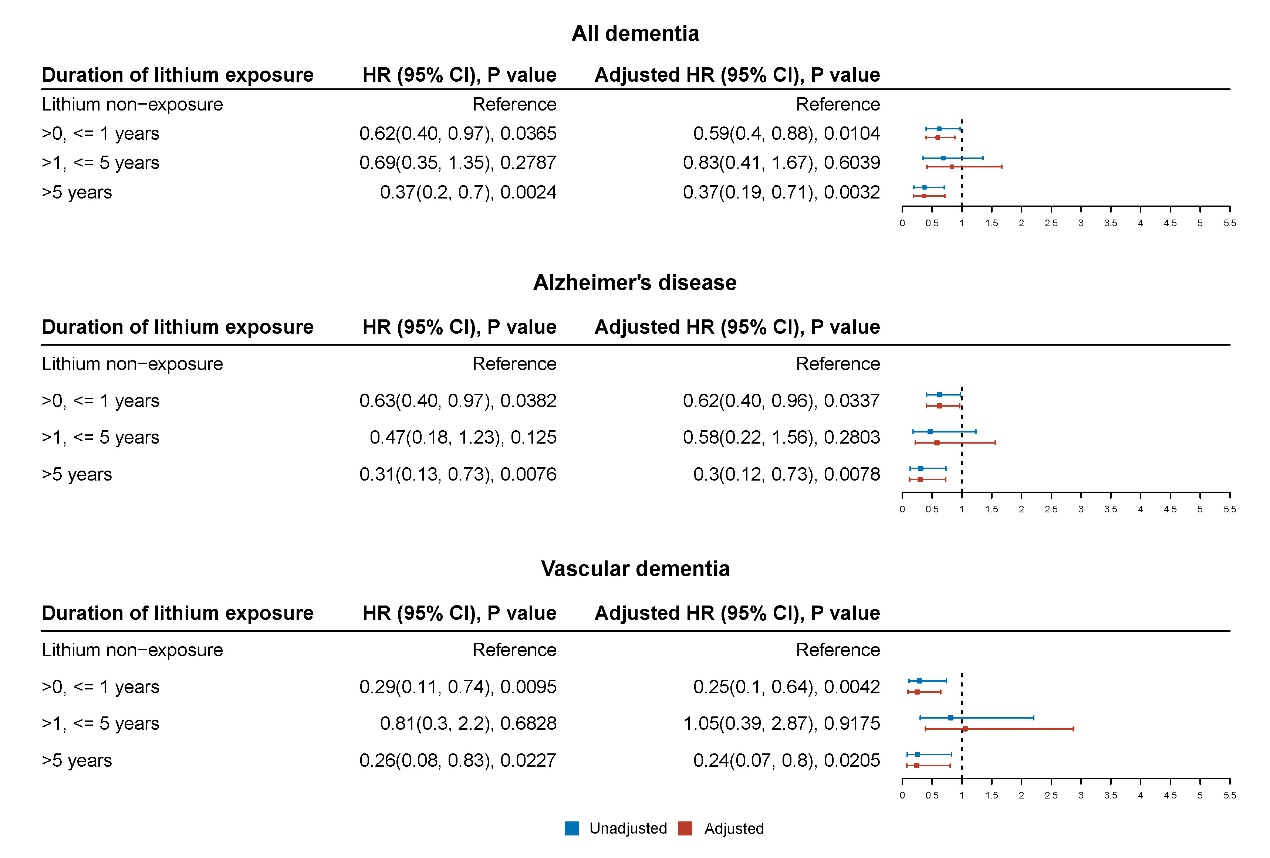


**Fig R. Association of duration of lithium exposure with the development of dementia and its subtypes by Cox proportional hazards models: sensitivity analysis by adding an interaction term between lithium exposure and exposure duration (in addition to the main effect of lithium exposure). Hazard ratios are for exposure duration (the interaction term); thus, HRs < 1 indicate a progressive reduction in the risk of dementia for each additional year of lithium exposure (over and above the fact of lithium exposure).** Unadjusted/adjusted hazard ratios (HRs), 95% confidence intervals (CI), and p values were extracted from inverse-probability-weighted Cox regression. Adjusted for age, sex, marital status, ethnicity, smoking status, alcohol disorders, antipsychotic use, depression, mania or bipolar affective disorder, hypertension, central vascular disease, diabetes mellitus, and hyperlipidaemias.


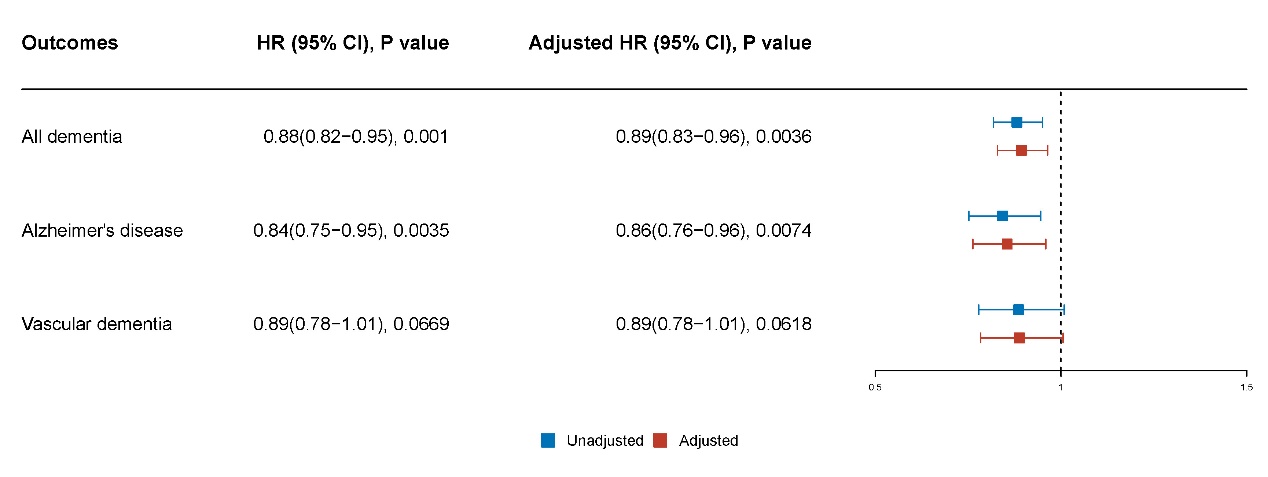

Supplement: S1 Appendix — Fig A: Association of lithium use with the development of dementia and its subtypes by Cox proportional hazards models: sensitivity analysis with a longer (2-year) criterion for identifying medications/comorbidities (see Methods). Adjusted HRs, 95% CIs, and p-values were extracted from inverse probability weighted Cox regression. Adjusted for age, sex, marital status, ethnicity, smoking status, alcohol disorders, antipsychotic use, depression, mania or BPAD, hypertension, central vascular disease, diabetes mellitus, and hyperlipidemias. Fig B: Association of duration of lithium exposure with the development of dementia and its subtypes by Cox proportional hazards models: sensitivity analysis with a longer (2-year) criterion for identifying medications/comorbidities (see Methods). Adjusted HRs, 95% CIs, and p-values were extracted from inverse probability weighted Cox regression. Adjusted for age, sex, marital status, ethnicity, smoking status, alcohol disorders, antipsychotic use, depression, mania or BPAD, hypertension, central vascular disease, diabetes mellitus, and hyperlipidemias. NA indicates no result (no corresponding cases). Fig C: Association of lithium use with the development of dementia and its subtypes by Cox proportional hazards models: sensitivity analysis requiring at least 2 years of follow-up. Adjusted HRs, 95% CIs, and p-values were extracted from inverse probability weighted Cox regression. Adjusted for age, sex, marital status, ethnicity, smoking status, alcohol disorders, antipsychotic use, depression, mania or BPAD, hypertension, central vascular disease, diabetes mellitus, and hyperlipidemias. Fig D: Association of duration of lithium exposure with the development of dementia and its subtypes by Cox proportional hazards models: sensitivity analysis requiring at least 2 years of follow-up. Adjusted HRs, 95% CIs, and p-values were extracted from inverse probability weighted Cox regression. Adjusted for age, sex, marital status, ethnicity, smok [file pmed.1003941.s002.docx]
